# From the Field to the Pot: Phytochemical and Functional Analyses of *Calendula officinalis* L. Flower for Incorporation in an Organic Yogurt

**DOI:** 10.3390/antiox8110559

**Published:** 2019-11-15

**Authors:** Graziela Bragueto Escher, Lorena do Carmo Cardoso Borges, Jânio Sousa Santos, Thiago Mendanha Cruz, Mariza Boscacci Marques, Mariana Araújo Vieira do Carmo, Luciana Azevedo, Marianna M. Furtado, Anderson S. Sant’Ana, Mingchun Wen, Liang Zhang, Daniel Granato

**Affiliations:** 1 Food Science and Technology Graduate Program, State University of Ponta Grossa, 84030-900 Ponta Grossa, Paraná, Brazil; santosjs.food@gmail.com; 2 Department of Food Engineering, State University of Ponta Grossa, 84030-900 Ponta Grossa, Paraná, Brazil; lorena.ccborges@hotmail.com; 3 Department of Chemistry, State University of Ponta Grossa, 84030-900 Ponta Grossa, Paraná, Brazil; mcruz.thiago01@gmail.com (T.M.C.); marizaboscacci@yahoo.com.br (M.B.M.); 4 Department of Biological Sciences, Federal University of Alfenas, 37130-000 Alfenas, Minas Gerais, Brazil; marianavieira06@hotmail.com (M.A.V.d.C.); lucianaazevedo2010@gmail.com (L.A.); 5 Department of Food Science, Faculty of Food Engineering, University of Campinas, 13083-862 Campinas, São Paulo, Brazil; marianna.mirandafurtado@gmail.com (M.M.F.); and@unicamp.br (A.S.S.); 6 State Key Laboratory of Tea Plant Biology and Utilization, Anhui Agricultural University, Hefei 230036, China; daniel.granato@luke.fi (M.W.); zhli2091@sina.com (L.Z.); 7 Food Processing and Quality, Innovative Food System, Production Systems Unit—Natural Resources Institute Finland (Luke)—Tietotie 2, FI-02150 Espoo, Finland

**Keywords:** antihemolytic effect, free radicals, antiproliferative activity, natural products, functional foods, edible flowers

## Abstract

Edible flowers have been used as ingredients because of their biological activities, taste, and overall appearance. This research was aimed to characterize the chemical composition and in vitro antioxidant activity of the marigold flower (*Calendula officinalis* L.) extracted with different proportions of water and ethyl alcohol, and the lyophilized extract with higher content of antioxidant compounds was incorporated into an organic yogurt. Results showed that the hydroalcoholic extract (50:50 v/v) presented the highest total phenolic content (TPC), flavonoids, and antioxidant activity (ferric reducing antioxidant power (FRAP), total reducing capacity (TRC), and Cu^2+^/Fe^2+^ chelating ability). Phenolic acids and flavonoids were quantified in the extract by LC-DAD, while 19 compounds were tentatively identified by ESI-MS/MS. The lyophilized marigold extract (LME) also inhibited 12% of Wistar rat’s brain lipid oxidation *in vitro*, inhibited α-amylase, and α-glucosidase activities, but showed no cytotoxicity towards cancerous cells (HCT8 and A549). However, marigold flower extract protected human erythrocytes against mechanical stress. When added into an organic yogurt model (0 to 1.5%), LME increased TPC and antioxidant activity (2,2-diphenyl-1-picrylhydrazyl (DPPH) and TRC), and the sensory analysis showed that the organic yogurt had an acceptance of 80.4%. Our results show that the use of LME may be a technological strategy to increase the content of bioactive compounds in yogurts.

## 1. Introduction

According to Kennedy [1], consumers are seeking out organic dairy foods because they are considered healthier and free of artificial conservatives. In addition, food safety, environmental protection, animal welfare, and an increase in the use of natural and organic products drive the market growth. Organic dairy foods are manufactured using organic milk, which is obtained from livestock using certified and standardized organic farming methods. Indeed, the continuous introduction of innovative organic dairy products drives the markets of organic milk-based foods [2]. The global organic dairy foods and drinks market are expected to reach $36,729 million by 2022 from $14,517 million in 2015 at a compound annual growth rate of 14.25% from 2016 to 2022 [3]. It is evident that new organic foods, especially dairies, are needed to increment the consumer’s expectations.

Yogurt is the most consumed dairy product worldwide, likely because of its nutritional, functional, and sensory properties. The yogurt contains bioactive peptides formed during milk fermentation with *Lactobacillus* strains that have antioxidant and antimutagenic properties [4]. At the same time, organic yogurt is considered superior to conventional yogurt in terms of health, respect for the environment, quality, and food safety [5]. In this regard, concerns about the safety of synthetic colorants and flavorings added in yogurt drive the replacement of natural colorings extracted from plants such as flowers, fruits, and vegetables [6,7].

Nowadays, edible flowers are gaining prominence for being a source of vitamins, minerals, fibers, and bioactive compounds that can be extracted by different methods [8]. Díaz-García et al. [9] found that the anthocyanin-rich extract of the flowers of *Thymus moroderi* added in yogurt kept the pink color stable for one month of storage. Granato et al. [10] emphasize that the use of herbal extracts in different dairy products confer antioxidant, antimicrobial, sensory, and can replace the synthetic coloring by natural dyes.

The flowers of *Calendula officinalis*, known as pot marigold, are used in traditional folk medicine in the preparation of aqueous extract, ointments, among others, especially for treating inflammations and wounds [11]. Jan et al. [12] reported that *C. officinalis* is an important medicinal plant with diverse phytochemicals and biological activities, such as antioxidant, anti-inflammatory, antibacterial, gastroprotective, and hepatoprotective. Among the phytochemicals present in flowers of *C. officinalis*, the triterpenes [13], flavonoids [13,14,15], phenolic acids [13,15], quinones and coumarins [16], and carotenoids [16,17] stand out. 

The use of edible flowers that are part of the Brazilian flora, especially *Calendula officinalis*, represents viable and innovative technological alternatives within the food sector. Therefore, the objective of the present study was to evaluate the chemical composition and antioxidant activity in vitro of *Calendula officinalis* flower extracts obtained with the mixture of non-toxic solvents (water and ethyl alcohol), select the extract with the highest content of antioxidant compounds, and add this lyophilized extract to organic yogurt. The phenolic composition measured by chromatographic techniques and the *in vitro* functional properties of the *C. officinalis* lyophilized flower extract were determined. Additionally, phenolic composition, antioxidant activity, proximal composition, physicochemical parameters, and sensory acceptance of organic yogurt with lyophilized extract of the *C. officinalis* flower were analyzed.

## 2. Materials and Methods 

### 2.1. Chemicals and Cell Lines

All the solutions were prepared with ultrapure water (Millipore, São Paulo, Brazil). Folin–Ciocalteu reagent, 2-thiobarbituric acid, 2,4,6-tripyridyl-s-triazine (TPTZ), 2,2-diphenyl-1-picrylhydrazyl (DPPH), iron chloride(III) hexahydrate, copper(II) sulfate pentahydrate, acetonitrile, high-performance liquid chromatography analytical standards (Gallic, syringic, 2-hydroxycinnamic, protocatechuic, 2,4-dihydroxybenzoic, 2,5-dihydroxybenzoic, *p*-coumaric, 5-*O*-caffeoylquinic, caffeic, ferulic, rosmarinic, and ellagic acids; quercetin-3-rutinoside, procyanidin A2, (−)-epicatechin, (+)-catechin, and quercetin), MTT (3-(4,5-dimethylthiazol-2-yl)-2,5-diphenyltetrazolium bromide), Dulbecco’s modified Eagle’s medium (DMEM)/low glucose, DMEM/Ham-F12, DCFH-DA (dichlorofluorescein diacetate), penicillin, and streptomycin were purchased from Sigma-Aldrich (São Paulo, Brazil). 2,4-Dihydroxybenzoic and 2,5-dihydroxybenzoic acids were purchased from Carl Roth (Karlsruhe, Germany). Ethyl alcohol 99.8%, methyl alcohol 99.8%, petroleum ether, ascorbic acid, and glacial acetic acid were purchased from Pró-Análise (Porto Alegre, Brazil). A549, HCT8, and IMR90 (healthy lung fibroblast cells) cell lines were obtained from the Rio de Janeiro cell bank (BCRJ, Rio de Janeiro, Brazil).

### 2.2. Vegetal Materials, Experimental Design, and Extraction

Organic dried flowers of *Calendula officinalis* L., certified by Group Ecocert Brazil (Florianópolis, SC, Brazil), were purchased from the Kampo industry (Ribeirão Preto, SP, Brazil) in August 2018. The flowers were ground and then standardized in a 60 Tyler mesh sieve. An exsiccate of *C. officinalis* flowers was deposited in the Herbarium of Medicinal Plants of State University of Ponta Grossa (UEPG) under number 22236.

To establish the ratio between water and ethyl alcohol (EtOH) with the best performance in the extraction of chemical compounds (β-carotene, total phenolic content, flavonoids, and *ortho*-diphenolics), instrumental color (color intensity, hue, yellow, red, and blue pigments) and *in vitro* antioxidant activity (DPPH, ferric reducing antioxidant power (FRAP), Folin–Ciocalteu reducing capacity, chelating activity against Cu^2+^ and Fe^2+^ ions) five extraction solutions were evaluated (water/EtOH in the following proportions [v/v]: 100/0, 75/25, 50/50, 25/75, 0/100). The five *C. officinalis* flower extracts were obtained randomly with a ratio of 1:15 (w/v). Extractions were performed with a controlled temperature of 60 ± 1 °C and a time of 30 min under constant magnetic stirring. The extracts were filtered and stored (8 °C) protected from light. 

### 2.3. Chemical Composition, Instrumental Color, and Antioxidant Activity of the Extracts

The total carotenoids (TC) of *C. officinalis* flower extracts were quantified according to the colorimetric method described by Rodriguez-Amaya [18], and the results were expressed in µg of β-carotene per 100 g of flowers. The total phenolic content (TPC) was determined by the Prussian Blue method [19], and the results were expressed in mg of gallic acid equivalents per 100 g of flowers. Total flavonoids (TF) were quantified by the UV-Vis spectrophotometric method (Shimadzu UV-1800, Kyoto, Japan) according to the methodology described by Lees et al. [20]. The TF content was expressed as mg quercetin equivalents per 100 g of flowers. The *ortho*-diphenolics were quantified according to Durán et al. [21], and the results were expressed in mg of chlorogenic acid equivalents per 100 g of flowers.

The instrumental color (IC) analyses were determined according to the methodology described by Glories [22]. The absorbances were recorded at wavelengths of 420, 520, and 620 nm in the UV-Vis spectrophotometer (Shimadzu UV-1800, Kyoto, Japan). Color intensity (CI), hue (H), and proportion of yellow (YP), red (RP), and blue (BP) pigments were calculated using Equations (1)–(5), respectively.

(1) $$\mathrm{Color}\;\mathrm{intensity}\;=\;Abs_{420\;nm}+\;Abs_{520\;nm}+\;Abs_{620\;nm}$$

(2) $$\mathrm{Hue}\;=\;\frac{Abs_{420\;nm}}{Abs_{520nm}}$$

(3) $$\%\;\mathrm{yellow}\;\mathrm{pigment}\;=\;\left(\frac{Abs_{420\;nm}}{CI}\right)\;\times 100$$

(4) $$\%\;\mathrm{red}\;\mathrm{pigment}\;=\;\left(\frac{Abs_{520\;nm}}{CI}\right)\;\times 100$$

(5) $$\%\;\mathrm{blue}\;\mathrm{pigment}\;=\;\left(\frac{Abs_{620\;nm}}{CI}\right)\;\times 100$$

The antioxidant activity (AA) by capturing the DPPH radical was analyzed according to Brand-Williams et al. [23], and ferric reducing antioxidant power (FRAP) was verified according to Benzie et al. [24]. The results of DPPH and FRAP were expressed in mg of ascorbic acid equivalents per 100 g of flowers. The reducing capacity of the Folin–Ciocalteu reagent (RCFC) was determined according to Singleton et al. [25], and the results were expressed in mg of gallic acid equivalents per 100 g of flowers. The chelating activity in relation to Cu^2+^ (CCA) and Fe^2+^ (FCA) ions was determined according to the experimental conditions described by Santos et al. [26], and the results were expressed as the percentage of inhibition of the formation of the Cu^2+^-pyrocatechol violet complex and Fe^2+^-ferrozine complex, respectively. All analyses were performed in triplicate.

### 2.4. Extract Selection by Principal Component Analysis 

The extract with higher chemical content, in vitro antioxidant activity, and color parameters was selected using principal component analysis (PCA). Firstly, the triplicate values were transformed into *z-*scores to standardize the results in unit variance. Subsequently, the correlation matrix of *Calendula officinalis* flower extracts (*n* = 15) in rows was elaborated, and the response variables (*n* = 14) in columns, totaling 210 data points [27]. The projection in the two-dimensional plane (PC1xPC2) was performed with the variables with higher load factors or equal to 0.60. Approximately 4 L of the selected extract were divided into 50 mL plastic containers, which were subjected to lyophilization under vacuum of 830 µmL Hg for 120 h (Terroni, model LD 1500A, São Carlos, Brazil).

### 2.5. Individual Phenolic Composition of the Selected Extract

The individual phenolic compounds from the hydroalcoholic extract (50:50 v/v) of *C. officinalis* flowers were quantified using high-performance liquid chromatography, HPLC (Shimadzu, model LC-20T Prominence, Kyoto, Japan) with photodiode array detector (DAD; model SPD-M20A, Shimadzu, Kyoto, Japan). Table 1 contains the chromatographic conditions for quantification of phenolic acids (*p*-coumaric, caffeic, ferulic, and ellagic) and flavonoids (procyanidin A2 and quercetin-3-rutinoside) quantified in the study. Chromatographic separation was performed using reversedphase and a C_18_ column (150 mm × 4.6 mm, particle size 3.5 μm; Agilent, Santa Clara, CA, USA). The mobile phase consisted of water acidified with 0.2% (v/v) formic acid (solvent A) and acetonitrile (solvent B). The elution gradient applied was 0 to 10 min 97% A, 10 to 15 min 90% A, 15 to 17 min 88% A, 17 to 23 min 45% A, 23 to 30 min 35% A, 30 to 35 min 100% A, and 35 to 47 min 100% A. Throughout the chromatographic separation the temperature of the column was maintained at 40 °C, and the sample injection volume was 10 μL with the flow rate of 0.5 mL/min. The quantification of phenolic compounds was performed by employing analytical curves, and the results were expressed in mg per 100 g of dried flowers, according to Fidelis et al. [28].

The qualitative identification of phenolic compounds from hydroalcoholic extract of *C. officinalis* flowers was performed using a mass spectrometry [ESI-MS/MS] Xevo^®^ (Waters, MA, USA). The negative ionization mode [M–H]^-^ was used, and the capillary and cone voltages were set at 30 V and 3 kV, respectively. The desolvation line temperature was 350 °C at 550 L/h (nitrogen gas flow) with collision energy of 20 V. The full scan mass covered the range from *m/z* 100 to 1300 and the scan time was 0.5 s (Table 2).

### 2.6. In Vitro Functional Properties of the Lyophilized Marigold Extract (LME)

The antimicrobial activity of LME was evaluated by the diffusion method in the agar-well plate as described by Cleeland et al. [31]. The standardized cell suspension (10^6^ CFU/mL) of *Pseudomonas aeruginosa* (IAL 1853), *Salmonella* Enteritidis (S 2887), *Salmonella* Typhimurium (IAL 2431), and *Escherichia coli* (IAL 2064) were grown in nutrient broth at 37 °C for 24 h; *Bacillus cereus* (ATCC 14579) and *Staphylococcus aureus* (ATCC 13565) were grown in nutrient broth at 30 °C for 24 h; *Listeria monocytogenes* (ATCC 7644) was grown in soy tryptone broth added with 0.6% yeast extract at 37 °C for 24 h; and *Saccharomyces cerevisiae* (NCYC 1006) was grown in malt extract broth at 30 °C for 24 h. The extract was dissolved with dimethyl sulfoxide (DMSO), and 2 mg added in each well (0.7 cm). The ampicillin (10 μg/mL) was used as a positive control, except for *S. cerevisiae* cicloheximide (1 μg/mL) was used, and sterile water as a negative control. The results were expressed as inhibition halo (cm).

The antihemolytic effect of LME was evaluated under hypotonic and isotonic conditions according to Zhang et al. [32]. The blood with O^+^ blood typing was obtained from the UEPG University Hospital, Ponta Grossa, Brazil, after approval by the Human Research Ethics Committee (CAAE: 94830318.1.0000.0105). The erythrocytes were isolated after successive washes with phosphate buffered saline (PBS, 5 mmol/L, pH 7.4, 154 mmol/L [0.9%] NaCl). The extract, 50 mg/L, was solubilized in PBS with 0.1–0.9% (w/v) NaCl concentrations and 0.8% hematocrit. The dose dependence of the extract, 50–110 mg/L, was evaluated under hypotonic conditions (PBS, 5 mmol/L, pH 7.4, 68 mmol/L [0.4%] NaCl). The results were expressed as a percentage of hemolysis.

The inhibition of lipid oxidation of the brain tissue of Wistar rats was evaluated according to the experimental conditions described by Migliorini et al. [33]. The UEPG Animal Research Ethics Committee approved process n° 047/2017 for the use of brain tissue from Wistar rats. The extract was solubilized in ultrapure water with a concentration of 1000 mg/L, and the quercetin standard (50 mg/L) was used as a positive control. The results were expressed as percentage inhibition of lipid oxidation.

The in vitro inhibition of α-amylase and α-glucosidase was assessed using the method adopted by Johnson et al. [34]. For that purpose, different concentrations of the extract were tested in triplicate (α-amylase: 1, 5, 10, 15, and 20 mg/mL; α-glucosidase: 50, 100, 200, 300, and 500 µg/mL). Acarbose (1 mmol/L) was used as a positive control for the α-amylase analyses and results were presented as inhibition (%).

The in vitro antiproliferative activity was evaluated for A549, HCT8, and IMR90 cells. The cell lines were maintained as described by Escher et al. [35]. Cells were plated into 96-well plates at a density of 1 × 10^4^ cells/well (A549 and HCT8), and 5 × 10^3^ (IMR90), 100 μL/well and they were treated with different concentrations (100–1000 μL/mL) of LME. The cell viability test was performed as proposed by Santos et al. [36], by the MTT assay. All experiments were carried out in quadruplicate, and the dose-response analysis was determined by non-linear regression. The IC_50_, GI_50_, and LC_50_ parameters were calculated per the method described by Carmo et al. [37].

The generation of intracellular ROS was measured by a ROS assay with DCFH-DA, as described by Escher et al. [35]. Briefly, cancerous A549 cells (6 × 10^4^ per well) and healthy IMR90 cells (2 × 10^4^ per well) were treated with different concentrations of lyophilized marigold extract (10, 50, and 100 μg/mL), 15 μmol/L hydrogen peroxide (positive control), or culture medium (negative control) diluted in DCFH-DA solution (25 mmol/L). Then, the cells were incubated at 37 °C for 1 h with the extract concentrations, and subsequently, they were washed with PBS and a H_2_O_2_ solution (15 μmol/L) was added. The ROS level was determined by fluorescence (excitation of 485 nm and emission of 538 nm), and results were reported as the percentage of fluorescence intensity.

### 2.7. Yogurt Manufacture and Analysis

Yogurts (~2 L per formulation) were prepared according to Perina et al. [38]. First, the organic whole bovine milk (Escher Organics, Campo Magro, Brazil) was added with 110 g/L organic sucrose (União, Barra Bonita, Brazil; IBD Certified), and the mixture was pasteurized at 65 °C/30 min. After cooling the mixture to 42 °C, 0.05 g/L of lyophilized bacterial culture (*Streptococcus salivarius* subsp. *thermophilus* and *Lactobacillus delbrueckii* subsp. *bulgaricus*) was added. The mixture was incubated at 42 °C until the clot point (pH 4.6). The curd was cooled to 7 °C, homogenized, and added at different concentrations of lyophilized marigold extract: 0.25, 0.50, 1.00, and 1.50 g/100 g yogurt. The negative control was elaborated with 0 g extract/100 g yogurt. The yogurts were stored at 7 °C until the time of analysis.

To determine the total phenolic content and antioxidant activity of yogurts, 5 g samples were extracted with 5 mL of methyl alcohol (1:1 v/v) and vortexed for 5 min. Subsequently, the mixture was centrifuged at 900× *g* for 15 min, and the upper phase layer collected and analyzed immediately. The TPC content (mg gallic acid equivalents (GAE)/100 g) and antioxidant activity by DPPH (mg AAE/100 g) were determined according to Margraf et al. [19] and Brand-Williams et al. [23], respectively. The total reducing capacity was measured using the Folin–Ciocalteu method modified by Berker et al. [39], and the results were expressed in mg quercetin equivalents per 100 g of yogurt.

A preliminary sensory analysis of the five yogurt formulations (data not shown) performed by untrained assessors indicated that lyophilized marigold extract (LME) concentrations above 0.25 g/100 g enhanced the bitter and astringent taste in yogurt, which is an undesirable feature of polyphenols. Therefore, the formulation containing 0.25 g LME/100 g yogurt was selected for the analysis of proximal composition, physicochemical parameters, instrumental texture, and sensory analysis.

The proximal composition of organic yogurt added at 0 g and 0.25 g LME/100 g was determined according to AOAC [40]: proteins (Nx6.38), total lipids (Bligh–Dyer method), moisture (105 °C/24 h), ashes (550 °C), and total carbohydrates was estimated by difference. The results were expressed in g/100 g of yogurt. The total caloric value was determined using Atwater factors (4 kcal/g for carbohydrates and proteins, and 9 kcal/g for lipids), and expressed in kcal/100 g of yogurt. The pH was measured at 25 °C in pH meter (Model HSP-3B, Labmeter, Brazil) previously calibrated. The total titratable acidity was verified using NaOH solution (0.1 mol/L), and the results were expressed as g lactic acid/100 g yogurt.

The instrumental color of yogurts (0 g and 0.25 g LME/100 g) using the MiniScan^®^ EZ 4500L colorimeter (Hunter Lab, Reston, USA) was expressed in scale parameters developed by the *Commission Internationale de Eclairage* (CIE): lightness (L*), green-red component (a* axis), and blue-yellow component (b* axis). The instrumental texture was determined using the Universal TA-XT2 Texture Analyzer (Stable Micro Systems, Surrey, UK). The double compression test with an aluminum cylinder (25 mm diameter) with a depth of 20 mm and a speed of 1 mm/s was used. The firmness (g) and consistency (g.s) parameters were measured by the Texture Expert v. 1.20 program using the algorithm Fracture TPA.

Sensory analysis with 76 untrained assessors (17 men and 59 women between 18 and 35 years) was carried out after signing the consent form approved by the Ethics Committee of the UEPG (approval code—CAAE: 65493717.9.0000.0105). The yogurt containing 0.25 g LME/100 g (20 g) was served in a glass identified with three random digits. A structured 9-point hedonic scale (1—extremely disliked to 9— extremely liked) was used to evaluate the degree of liking of odor, taste, texture, color, and overall impression. The assessors were asked if they consider yogurt a healthy product (yes/no). Moreover, the assessors opined on the overpayment (R$ 0 to R$ 3.00) for a 100 g unit of “yogurt rich in natural antioxidant compounds” and “organic yogurt” compared to “yogurt without antioxidant” and “conventional yogurt” (R$ 1.00), respectively.

### 2.8. Statistical Analyses

The results were expressed as mean ± standard deviation (*n* = 3). The Brown–Forsythe test was applied to evaluate the homoscedasticity of the data. The one-way analysis of variance (ANOVA) was applied to evaluate the differences between the mean values, followed by Fisher’s least significant difference test (*p* ≤ 0.05). The linear correlations between the results (*n* = 15) were evaluated by Pearson’s correlation coefficient (*p* ≤ 0.05). The results of antimicrobial activity, lipid oxidation inhibitory activity, proximal composition, physicochemical parameters, and instrumental texture were evaluated by unpaired Student *t* test (*p* ≤ 0.05). The TIBCO Statistica software v. 13.3 (TIBCO Statistica™ Ltd., Palo Alto, CA, USA) was used in the analyses.

## 3. Results and Discussion 

### 3.1. Chemical Composition, Instrumental Color, and Antioxidant Activity of the Extracts

Table 3 shows the chemical composition and instrumental color of *C. officinalis* flower extracts. The β-carotene content ranged from 97 to 637 µg/100 g (*p* ≤ 0.05). The highest content of β-carotene was extracted with 100% EtOH, and significant differences were not observed between extracts with 50 and 75% EtOH. Carotenoid contents ranging from 200 to 3510 mg/100 g were quantified in different *C. officinalis* flower varieties [17,41]. The marigold flowers as well as β-carotene containing α-carotene, γ-carotene, lutein, luteoxanthin, flavoxanthin, rubixanthin, and other carotenoids [42,43]. 

The TPC contents ranged from 123 to 217 mg GAE/100 g (*p* ≤ 0.05). The highest TPC contents were extracted with 50% H_2_O:50% EtOH, followed by 75% H_2_O:25% EtOH and 25% H_2_O:75% EtOH. The TF contents ranged from 101 to 147 mg QE/100 g (*p* ≤ 0.05), and the highest content was extracted with 50% H_2_O:50% EtOH. The *ortho*-diphenolics content ranged from 331 to 531 mg CAE/100g (*p* ≤ 0.05). The highest *ortho*-diphenolics content was extracted with 25% H_2_O:75% EtOH, and significant differences were not observed between the extracts obtained with 50% H_2_O:50% EtOH and 100% EtOH.

Ferreira et al. [15] compared chloroform, ethyl alcohol, methyl alcohol (MeOH), and hydromethanolic (MeOH:H_2_O, 70:30 v/v) solvents in the extraction of phenolic compounds from marigold flowers. The authors found that hydromethane was more effective in extracting phenolic compounds because it was highly polar. Pires et al. [44] quantified in the extracts of marigold petals containing MeOH:H_2_O (80:20 v/v) the contents of 1131 mg/100 g of phenolic compounds and 1115 mg/100 g of flavonoids, and in the aqueous extract the contents of 747 mg/100 g and 737 mg/100 g, respectively. The literature shows that methyl alcohol and hydromethanolic solutions extract higher levels of phenolic compounds. However, in this research, it was chosen to use ethyl alcohol because of its low toxicity. In addition, the contents of bioactive compounds (carotenoids and phenolic compounds) may vary due to different *C. officinalis* flower varieties and cultivation sites [41,45]. 

The color indices of the extracts were influenced by the combination of the solvents H_2_O and EtOH in which the color intensity varied from 1.1 to 2.1 u. a. and the hue ranged from 4.9 to 12.6 u. a. (*p* ≤ 0.05). The highest intensity of color and hue was verified in the extract obtained with 100% EtOH which presented the highest content of β-carotene (637 µg/100 g). According to Khalid et al. [45], carotenoids are mainly responsible for the yellow-orange coloration of *C. officinalis* inflorescences. In the correlation analysis, color intensity was significantly correlated (*p* ≤ 0.05) with β-carotene (*r* = 0.8959), *ortho*-diphenolics (*r* = 0.6150), and TPC (*r* = −0.5393). The hue showed a significant correlation (*p* ≤ 0.05) with β-carotene (*r* = 0.7874) and TPC (*r* = −0.8702).

The yellow, red, and blue pigments of the extracts ranged from 79 to 90%, 7 to 16%, and 3 to 6% (*p* ≤ 0.05), respectively. The highest percentage of yellow pigments (90%) was obtained in the extract with 100% EtOH. Higher percentages of red (16%) and blue (6%) pigments were obtained in extracts with 75% H_2_O:25% EtOH and 100% H_2_O, respectively. The yellow pigments showed significant correlation (*p* ≤ 0.05) with β-carotene (*r* = 0.7765) and TPC (*r* = −0.7516). The red and blue pigments correlated significantly (*p* ≤ 0.05) with β-carotene, *r* = −0.7619 and *r* = −0.6724, respectively. The blue pigments also correlated with *ortho*-diphenolic (*r* = −0.5387), and the red pigment with TPC (*r* = 0.7909). Olennikov et al. [14] quantified anthocyanins in some *C. officinalis* varieties with intense raspberry-red pigmentation in the central part of the flower.

The chemical antioxidant activity of the extracts was influenced by the different proportions of the solvents H_2_O and EtOH (*p* ≤ 0.05) (Table 4). The extract obtained with 75% H_2_O:25% EtOH showed higher antioxidant activity through electron transfer evaluated by DPPH, FRAP, and RCFC. In the analysis by FRAP and RCFC, no significant differences were observed between the extracts with 75% H_2_O:25% EtOH and 50% H_2_O:50% EtOH. Additionally, the metal chelating activity of the extracts was evaluated by inhibiting the formation of the Cu^2+^-pyrocatechol violet complex and the Fe^2+^-ferrozine complex. The extract with 50% H_2_O:50% EtOH showed the highest chelating activity with inhibition of 60% and 53% of the formation of the Cu^2+^-pyrocatechol violet complex and Fe^2+^-ferrozine complex, respectively.

The antioxidant activity of the extract with 50% H_2_O:50% EtOH is associated with higher levels of TPC (217 mg GAE/100 g) and TF (QE 147 mg/100 g). *C. officinalis* flower extracts showed significant correlation (*p* ≤ 0.05) of antioxidant activity with β-carotene (r_DPPH_ = −0.7911; r_FRAP_ = −0.6637; r_CCA_ = −0.6479), total phenolic content (r_DPPH_ = 0.9109; r_FRAP_ = 0.9677; r_RCFC_ = 0.9663; r_CCA_ = 0.9279), flavonoids (r_FRAP_ = 0.5532; r_RCFC_ = 0.7154; r_CCA_ = 0.5341; r_FCA_ = 0.6371), and with *ortho*-diphenolics (r_FCA_ = 0.5479).

### 3.2. Principal Component Analysis (PCA)

**Figure 1:**
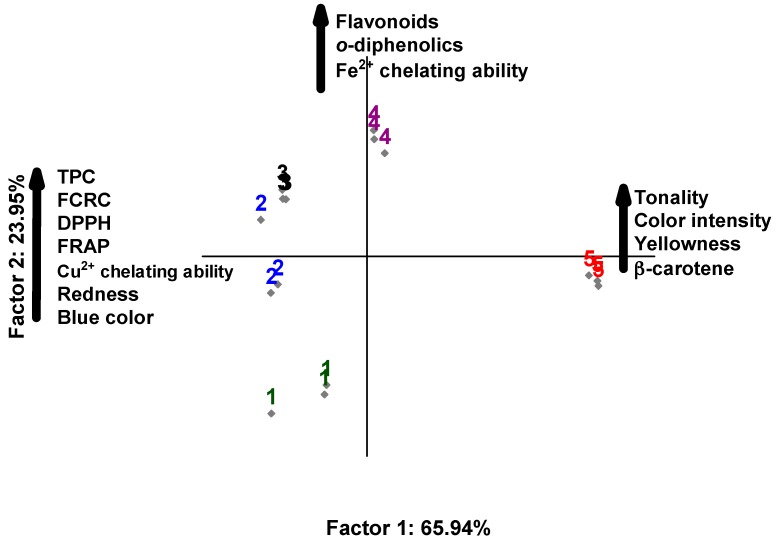
Principal component analysis based on physicochemical properties and antioxidant activity of marigold flower extracts (*Calendula officinalis* L.). Extract 1 = 100% H_2_O:0% EtOH; extract 2 = 75% H_2_O:25% EtOH; extract 3 = 50% H_2_O:50% EtOH; extract 4 = 25% H_2_O:75% EtOH; and extract 5 = 0% H_2_O:100% EtOH. TPC = total phenolic content; FCRC = Folin–Ciocalteau reducing capacity.

In the principal component analysis, the relationship of extracts with chemical compounds, instrumental color, and antioxidant activity was explored. The two-dimensional projection explained 90% of the data variability, and the main components 1 and 2 explained 65.94% and 23.95% of the data variability, respectively (Figure 1). The main component 1 showed that the extracts with 75% H_2_O:25% EtOH (assay 2) and 50% H_2_O:50% EtOH (assay 3) showed higher content of TPC, RP, YP, and antioxidant activity by DPPH, FRAP, RCFC, and CCA. The extract with 100% EtOH (assay 5) showed higher hue, color intensity, yellow pigment, and β-carotene content, but low antioxidant activity. In the principal component 2, the 25% H_2_O:75% EtOH extract (assay 4) correlated with flavonoids, *ortho*-diphenolics, and Fe^2+^ chelating activity, while the 100% H_2_O extract (assay 1) showed no correlation with chemical compounds, color parameters, and antioxidant activity. The PCA indicated that the extract with 50% H_2_O:50% EtOH showed higher values of antioxidant activity and bioactive compounds concerning the other extracts. Therefore, the extract obtained with 50% H_2_O:50% EtOH was selected for the analysis of the individual phenolic composition, *in vitro* functional properties, and incorporation in organic yogurt. 

### 3.3. Selected Extract: Individual Phenolic Composition and in Vitro Functional Properties

The individual phenolic composition of the hydroalcoholic extract (50:50 v/v) of *C. officinalis* flowers were determined by HPLC-DAD (Figure S1). The phenolic acids quantified were *p*-coumaric (5.8 mg/100 g), caffeic (9.2 mg/100 g), ferulic (18.3 mg/100 g), and ellagic acid, 3.7 mg/100 g. The quantified flavonoids were procyanidin A2 (42.5 mg/100 g) and quercetin-3-rutinoside (46.1 mg/100 g). Olennikov et al. [17] quantified caffeic acid (92 mg/100 g) and quercetin-3-rutinoside (226 mg/100 g) in the hydroalcoholic extract, 60% EtOH, of *C. officinalis* flowers var. Greenheart Orange. Pires et al. [44] quantified caffeic acid (1 mg/100 g) and quercetin-3-rutinoside (30 mg/100 g) in the hydromethanolic extract (MeOH:H_2_O, 80:20 v/v) of *C. officinalis* petals. Miguel et al. [29] quantified 22 mg/100 g quercetin-3-rutinoside in the hydromethanolic extract (MeOH:H_2_O, 80:20 v/v) of *C. officinalis* flowers.

Nineteen phenolic compounds were tentatively identified in the hydroalcoholic extract (50:50 v/v) of *C. officinalis* flowers by ESI-MS/MS ([M-H]^-^): caffeic acid hexoside (*m/z* 341), isomeric form of hydroxyferulic acid hexoside (*m/z* 371), caffeoylshikimic acid (*m/z* 335), 5-*O*-feruloylquinic acid (*m/z* 367), quercetin derivative (*m/z* 505; *m/z* 463; and *m/z* 609), quercetin-3-*O*-malonylhexoside (*m/z* 549), apigenin derivative (*m/z* 563), quercetin dihexoside (*m/z* 625), ligstroside hexoside (*m/z* 685), isorhamnetin derivative (*m/z* 477; *m/z* 519; and *m/z* 623), kaempferol-rhammosyrutinoside (*m/z* 739), quercetin-3-*o*-rhamnosylrutinoside (*m/z* 755), calenduloside G (*m/z* 793), calendasaponin B (*m/z* 971), and calendasaponin A (*m/z* 1117). The obtained mass spectra are shown in Figure S2. The compounds tentatively identified in this study corroborate data obtained by Miguel et al. [29] and Faustino et al. [30].

The antimicrobial activity of the lyophilized marigold extract is presented in Table 5. The extract showed the inhibitory effect of the growth of *L. monocytogenes*, *P. aeruginosa*, *S.* Typhimurium, *S.* Enteritidis, *B. cereus*, *E. coli*, and *S. aureus* (0.27 cm). These results corroborate Efstratiou et al. [46], who found that the ethanolic extract of *C. officinalis* petals inhibited the growth of *P. aeruginosa* (0.7 cm), *B. cereus* (1 cm), *E. coli* (0.3 cm), and *S. aureus* (2.2 cm). The hydromethanolic extract (MeOH:H_2_O, 80:20 v/v) and the infusion of petals *C. officinalis* demonstrated inhibitory effect against Gram-positive and Gram-negative bacteria [44]. According to Faustino et al. [30] methanolic extract of some *C. officinalis* varieties showed low antibacterial activity due to the lower content of antibacterial phenolic compounds such as caffeic acid.

**Figure 2:**
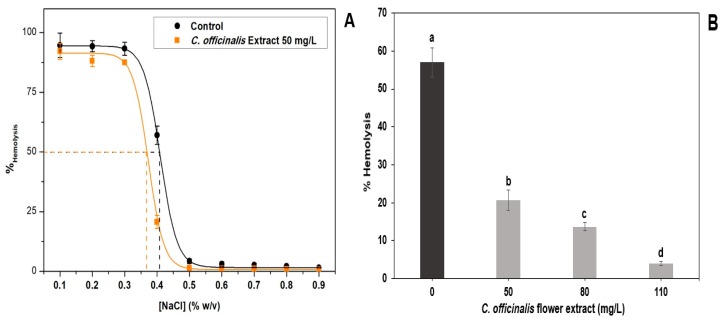
*In vitro* antihemolytic effect of lyophilized marigold extract (*C. officinalis*). (**A**) Effects on hemolysis of human erythrocytes at different concentrations of NaCl; (**B**) effects of different extract concentrations on hemolysis of human erythrocytes at 0.3% (w/v) NaCl. Different letters (a–d) represent statistically significant differences, *p* < 0.0001 (**B**).

The antihemolytic effect of LME analyzed under hypotonic and isotonic conditions is shown in Figure 2. Figure 2A shows that using the 50 mg/L of the LME, it was possible to calculate the H_50_, being the NaCl concentration where 50% of the erythrocytes is present in the sample are lysed. The lower this value, the more efficient the extract will be in inhibiting hemolysis caused by low extracellular osmotic pressure. The control, in the absence of the extract, had an H_50_ value of 0.413%, while the extract had a lower H_50_ value (0.356%). These results suggest that the bioactive compounds present in LME may interact with erythrocyte membrane phospholipids in order to reduce the flow parameter of the lipid bilayer [47], which increases the resistance of cells to hypotonic hemolysis [48].

Figure 2B shows that the hemolysis rate is reduced, 0.4% NaCl, with increasing LME concentration (50–110 mg/L). The *C. officinalis* flowers are rich in saponins, compounds that have hemolytic activity by solubilizing membranes [48]. The results showed that the extract showed antihemolytic activity, indicating the absence or low content of saponins in *C. officinalis* flowers. The antihemolytic activity of *C. officinalis* extracts had already been tested in the literature under oxidative and thermal stress conditions and, in both cases, the hydroalcoholic extract (EtOH:H_2_O, 80:20 v/v) was efficient in inhibiting hemolysis [49].

**Figure 3:**
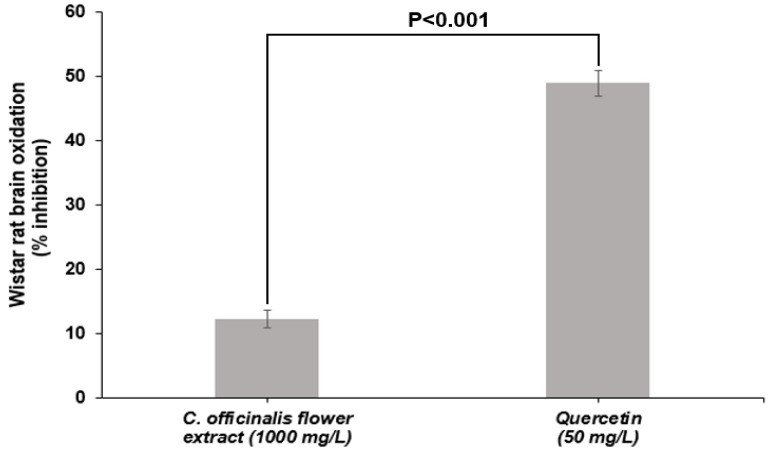
Inhibition of lipid oxidation of Wistar rat brain treated with lyophilized marigold extract (*C. officinalis*) in comparison to quercetin. Probability value was obtained using unpaired Student *t* text.

LME showed 12% inhibition of lipid oxidation of Wistar rat brains at a concentration of 1000 mg/L, statistically differing (*p* < 0.001) from quercetin inhibition capacity (50 mg/L) (Figure 3). The superiority of quercetin was already expected since it is a standard of high purity and with a recognized ability to inhibit lipid oxidation. However, the inhibitory capacity presented by the LME is an essential feature because it shows that it is active in biological media inhibiting the oxidation of brain tissue lipids. This test shows the ability of the compounds present in the extract to transfer H^+^ atom to free radicals generated by lipid-induced oxidation [50]. The ability of *C. officinalis* extracts to inhibit lipid oxidation was also observed in an in vivo study by Tanideh et al. [51]. The same authors found that the hydroalcoholic extract (EtOH:H_2_O, 80:20 v/v) of *C. officinalis* flowers, administered orally to male Sprague–Dawley rats, showed decreased lipid oxidation.

**Figure 4:**
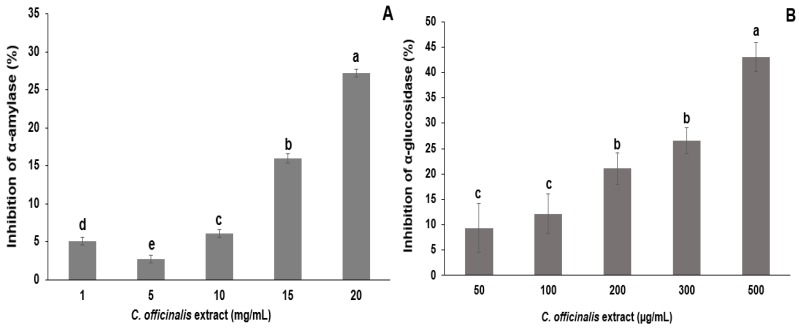
*In vitro* inhibitory effects of different concentrations of lyophilized marigold extract (*C. officinalis*) on the activities of α-amylase (**A**) and α-glucosidase (**B**) enzymes. Different letters (a–e) represent statistically significant differences (*p* < 0.0001).

LME, 20 mg/mL, inhibited 27% of α-amylase enzymatic activity, indicating a dose-dependent effect with increasing extract concentration (1–15 mg/mL) (Figure 4A). The LME, 500 µg/mL, inhibited 43% of α-glucosidase enzymatic activity. The concentrations of 50 to 100 µg/mL and 200 to 300 µg/mL of LME showed no significant differences in the inhibitory activity of α-glucosidase (Figure 4). The extract from the leaves of *C. officinalis* was evaluated in the inhibitory activity of the α-amylase enzyme as described by Olennikov et al. [52]. The authors found that 38.02 µg/mL of hydroalcoholic extract (EtOH:H_2_O, 60:40 v/v) of leaves inhibited 50% of α-amylase activity. In another study, hydroalcoholic extract (50:50 v/v) from *C. officinalis* leaves reduced serum glucose levels in diabetic rats [53].

**Figure 5:**
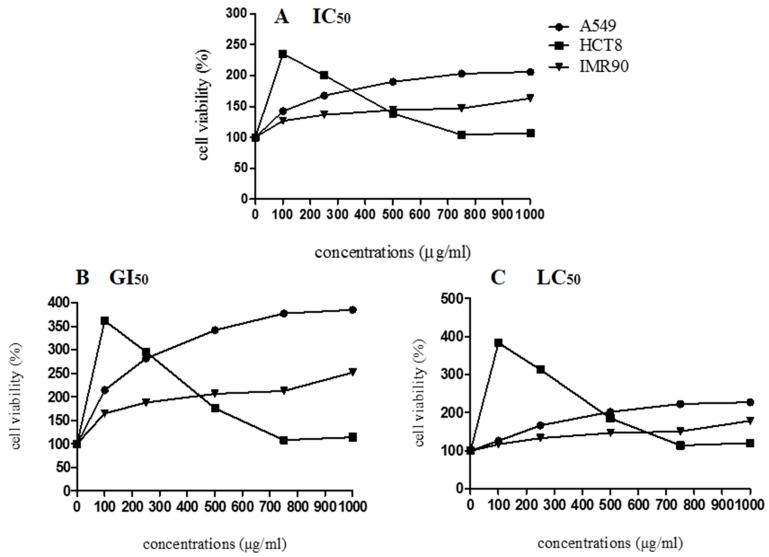
Cell viability of A549, HCT8, and IMR90 cells in relation to different concentrations of lyophilized marigold extract (*C. officinalis*). (**A**) IC_50_: The concentration of the agent that inhibits growth by 50% is the concentration at which (T/C) × 100 = 50, where T = number of cells, at time t of treatment; C = control cells at time t of treatment. (**B**) GI_50_: The concentration of the agent that inhibits growth by 50%, relative to untreated cells, is the concentration at which ([T−T0]/[C−[]T0]) × 100 = 50, where T and C are the number of treated and control cells, respectively, at time t of treatment and T > T0; T0 is the number of cells at time zero. (**C**) LC_50_: The concentration of the agent that results in a net loss of 50% cells, relative to the number at the start of treatment, is the concentration at which ([T−T0]/T0) × 100 = −50; T < T0.

According to the cytotoxicity results (Figure 5), the lyophilized marigold extract enhanced the cell viability parameter (IC_50_), stimulated the growth index (GI_50_), and it was not able to cause cell death (LC_50_) in both cancerous (HCT8, A549) and noncancerous (IMR90) cell lines, suggesting no apparent cytotoxicity. Similarly, Pires et al. [44] also observed that not all the *C. officinalis* samples inhibited the growth of MCF-7, NCI-H460, HeLa, and HepG2 tumor cells. Herein, considering the cell proliferation behavior, exogenous antioxidants (i.e., flavonoids and carotenoids) may exert cytotoxic effects in several cancerous cell lines, but whether an antioxidant supplement would be helpful, harmful, or neutral depends on the specific antioxidant, its dose, the chemotherapy drugs being used, and the type and stage of cancer [54]. Regarding the growth of A549, IMR90, and HCT8 cells, we hypothesize that the bioactive compounds in *C. officinalis* may favor mitogenic mechanisms, which involves complex pathways that can be related to overexpression of essential cell cycle regulatory proteins, such as cyclin-dependent kinases, d-type cyclins, polo-like kinases, and aurora kinases, which stimulate the cell cycle progression in the M/G1/S phases [55]. Bearing these concerns, more research is needed to explain the possible mechanisms involved in *C. officinalis*-induced cell growth.

**Figure 6:**
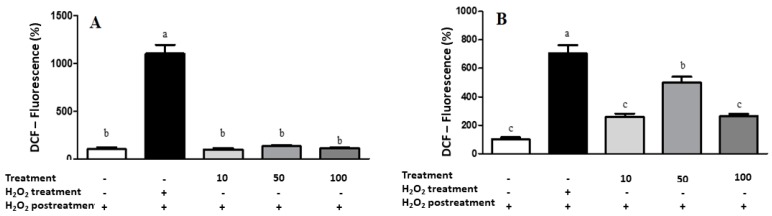
Results of intracellular ROS measurement in A549 (**A**) and IMR90 (**B**) cells by spectrofluoremetry. Treatment = lyophilized marigold extract (*C. officinalis*) at 10–100 μg/mL. Quantitative data are the mean ± standard deviation. Different letters (a–c) comparing the treatments indicate significant differences (*p* < 0.05).

Regarding the cell-based antioxidant assay, LME did not induce ROS generation (Figure 6) at the higher tested concentrations in both cancerous (A549) and noncancerous (IMR90) cells. Palozza et al. [56] observed that carotenoids in the *C. officinalis* extract have already been associated with antiproliferative activity and cytotoxicity through their prooxidant ability. Thus, although the *C. officinalis* extract presented an interesting chemical profile, antioxidant, antimicrobial, and anti-hemolytic capacities, it did not exhibit cytotoxic effects against the cancerous cell lines.

### 3.4. Characterization of the Organic Yogurt with LME

The TPC and antioxidant activity (DPPH and total reducing capacity (TRC)) of organic yogurts manufactured with different concentrations of LME are shown in Table 6. Increasing LME concentration, 0.25 to 1.5 g/100 g, increased the TPC and *in vitro* antioxidant activity of organic yogurts, and thus indicating a dose-dependence effect. These results corroborate with Demirkol et al. [57] who determined an increase in TPC (20 to 52 mg/100 g yogurt) and DPPH value (263 to 1100 mg/mL for 50% inhibition of the radical) in yogurt added with grape pomace (1 to 5 g/100 g milk). Abdel-Hamid et al. [58] measured an increase in antioxidant activity using the DPPH (43 to 78% radical inhibition) and ABTS (54 to 91% radical inhibition) assays in probiotic yogurt supplemented with 0.5 to 2% of *Siraitia grosvenorii* fruit extract.

The proximal composition, physicochemical characterization, color, and instrumental texture of organic yogurt with LME (0.25 g LME/100 g) and without extract (0 g LME/100 g, control) is presented in Table 7. The protein, lipid, ash content, titratable acidity, hardness, and consistency of organic yogurt manufactured with LME did not show statistically significant differences with the control. The organic yogurts with and without LME show statistically significant differences (*p* ≤ 0.05) in carbohydrate content, moisture, energy, and pH. These results are similar to those found by Karnopp et al. [2] in organic yogurt manufactured with Bordeaux grape skin flour. The same authors found the following properties in the yogurt: carbohydrate (9.35 g/100 g), protein (3.80 g/100 g), lipid (6.23 g/100 g), ash (0.81 g/100 g), moisture (76.49 g/100 g) contents, pH (4.19), titratable acidity (0.61 g lactic acid/100 g), hardness (20.88 g), and consistency (493.13 g/s). The incorporation of the LME in the organic yogurt caused a color change (L*, a*, and b*) compared to the control (*p* < 0.001) (Table 7). Demirkol et al. [57] also found a reduction in the brightness (L*) and increased redness (a*) in yogurt manufactured with grape pomace flour. According to Pires et al. [6], edible flower petal extracts have great potential for use as natural colorants and are an excellent source of bioactive compounds to develop innovative products with novel sensory characteristics and antioxidant activity.

The organic yogurt with 0.25 g LME/100 g presented a general sensory acceptance rate of 80.4%. The average scores given for odor (7.6 ± 1.5), taste (6.4 ± 2.2), consistency (7.2 ± 2.0), color (7.7 ± 1.5), and overall impression (7.2 ± 1.7) were ranked between “slightly liked” and “moderately liked “. The bitter residual taste reported by some assessors may have influenced the taste attribute (average grade <7). All evaluators (100%, *n* = 76) consider yogurt a healthy product. Regarding the commercial value of the yogurt, 75% of assessors would pay between R$ 2.00 and R$ 3.00 for yogurt rich in natural antioxidant compounds (100 g) compared to yogurt without natural antioxidants, and 75% would pay between R$ 2.00 and R$ 3.00 for organic yogurt (100 g) compared to conventional yogurt. 

## 4. Conclusions

The hydroalcoholic extract (50:50 v/v) of *Calendula officinalis* flowers presented the highest total phenolic content (TPC), flavonoids, and in vitro antioxidant activity (FRAP, TRC, and Cu^2+^/Fe^2+^ chelating ability). Quercetin-3-rutinoside, procyanidin A2, ferulic, caffeic, *p*-coumaric, and ellagic acids were quantified in the extract by LC-DAD, while 19 compounds were tentatively identified by ESI-MS/MS. The lyophilized marigold extract presented antimicrobial activity, antihemolytic activity, inhibition of lipid oxidation of Wistar rat’s brain, and inhibited α-amylase/α-glucosidase activities. The LME showed no cytotoxicity towards cancerous (A549 and HCT8) and noncancerous (IMR90) cells, and it did not induce intracellular ROS generation. The incorporation of different concentrations of LME in organic yogurt increased the total phenolic content and antioxidant activity. The organic yogurt with 0.25 g LME/100 g had a high acceptability index, demonstrating that the use of LME may be a technological and functional-led strategy to increase the content of bioactive compounds in yogurts.

## Figures and Tables

**Table 1:** Chromatographic parameters of phenolic compounds analyzed in the marigold extract by HPLC-DAD.

Compound	Retention Time (min)	Wavelength (nm)	Linear Range (mg/L)	LOD (mg/L)	LOQ (mg/L)	Analytical Curve	*R* ^2^
Caffeic acid	26.66	325	0.5–100	0.39	1.21	*y* = 105042*x* − 53002	0.9968
*p*-Coumaric acid	29.68	318	0.5–105	0.02	0.05	*y* = 124348*x* + 142935	0.9951
Ferulic acid	30.15	325	0.5–99	0.14	0.41	*y* = 105233*x* − 17124	0.9991
Procyanidin A2	17.70	280	0.5–92	1.76	5.34	*y* = 15356*x* + 8200.1	0.9996
Quercetin-3-rutinoside	17.52	360	0.5–114	0.01	0.02	*y* = 35743*x* − 1027.7	0.9995
Ellagic acid	17.52	360	0.5–99	0.07	0.20	*y* = 42138*x* − 8717.5	0.9991

Note: LOD = limit of detection, LOQ = limit of quantification.

**Table 2:** Mass spectral data and tentative identification of phenolic compounds in the hydroalcoholic extract of *Calendula officinalis* flowers according to the literature.

Compound	Molecular Ion [M−H]^−^(*m/z*)	MS (*m/z*)	Tentative Identification	Reference
1	341	179(100), 161(3), 135(40)	Caffeic acid hexoside	Miguel et al. [29]
2	371	353(20), 209(100), 191(18)	Isomeric form of hydroxyferulic acid hexoside	Faustino et al. [30]
3	335	291(4), 179(100), 173(30), 135(18)	Caffeoylshikimic acid	Faustino et al. [30]
4	367	193(14), 191(100), 173(32)	5-*O-*feruloylquinic acid	Faustino et al. [30]
5	505	301(65)	Quercetin derivative	Miguel et al. [29]
6	549	505(100), 387, 301	Quercetin-3-*O*-malonylhexoside	Faustino et al. [30]
7	563	545(2), 473(75), 353(26), 269(3)	Apigenin derivative	Faustino et al. [30]
8	463	301(100)	Quercetin derivative	Faustino et al. [30]
9	609	301(100)	Quercetin derivative	Miguel et al. [29]
10	625	463, 301	Quercetin dihexoside	Faustino et al. [30]
11	685	667(30), 523(100)	Ligstroside hexoside	Faustino et al. [30]
12	477	315(100), 300(15)	Isorhamnetin derivative	Miguel et al. [29]
13	519	315(100), 300(37)	Isorhamnetin derivative	Miguel et al. [29]
14	739	285(100)	Kaempferol-rhammosyrutinoside	Miguel et al. [29]
15	755	301(100)	Quercetin-3-*O*-rhamnosylrutinoside	Miguel et al. [29]
16	623	315(100)	Isorhamnetin derivative	Miguel et al. [29]
17	793	673(12), 631(100), 613 (20)	Calenduloside G	Faustino et al. [30]
18	971	971(100), 809(20), 471(15)	Calendasaponin B	Faustino et al. [30]
19	1117	1117(100), 955(10), 793(6)	Calendasaponin A	Faustino et al. [30]

**Table 3:** Chemical composition and instrumental color of *Calendula officinalis* flower extracts.

Assay	H_2_O (%)	EtOH (%)	β-Carotene (µg/100 g)	Total Phenolic Content (mg GAE/100 g)	Flavonoids (mg QE/100 g)	*Ortho*-Diphenolics (mg CAE/100 g)	Color Intensity (u. a.)	Tonality (u. a.)	Yellow Pigments (%, λ = 420 nm)	Red Pigments (%, λ = 520 nm)	Blue Pigments (%, λ = 620 nm)
1	100	0	97 ± 64 ^d^	180 ± 2 ^c^	101 ± 0 ^e^	331 ± 9 ^d^	1.3 ± 0.1 ^d^	5.5 ± 0.8 ^c^	79 ± 3 ^c^	14 ± 2 ^b^	6 ± 1 ^a^
2	75	25	205 ± 35 ^c^	207 ± 3 ^b^	120 ± 0 ^c^	394 ± 4 ^c^	1.1 ± 0.1 ^e^	4.9 ± 0.1 ^c^	79 ± 1 ^c^	16 ± 1 ^a,b^	4 ± 1 ^b^
3	50	50	429 ± 35 ^b^	217 ± 3 ^a^	147 ± 0 ^a^	425 ± 6 ^b^	1.6 ± 0.1 ^c^	5.2 ± 0.1 ^c^	79 ± 1 ^c^	15 ± 1 ^a^	5 ± 1 ^a^
4	25	75	409 ± 18 ^b^	206 ± 6 ^b^	141 ± 0 ^b^	531 ± 24 ^a^	1.9 ± 0.1 ^b^	7.7 ± 0.1 ^b^	85 ± 1 ^b^	11 ± 1 ^c^	4 ± 1 ^b^
5	0	100	637 ± 23 ^a^	123 ± 4 ^d^	105 ± 1 ^d^	425 ± 9 ^b^	2.1 ± 0.1 ^a^	12.6 ± 0.1 ^a^	90 ± 1 ^a^	7 ± 1 ^d^	3 ± 1 ^c^
*p-*Value (homoscedasticity)			0.549	0.880	0.510	0.373	0.189	0.123	0.119	0.165	0.068
*p-*Value (one-way ANOVA)			<0.001	<0.001	<0.001	<0.001	<0.001	<0.001	<0.001	<0.001	<0.001

Different letters (^a–d^) in the same column represent statistically significant results (*p* ≤ 0.05). GAE = gallic acid equivalents; QE = quercetin equivalents; CAE = chlorogenic acid equivalents.

**Table 4:** Antioxidant activity of *Calendula officinalis* flower extracts.

Assay	H_2_O (%)	EtOH (%)	DPPH (mg AAE/100 g)	FRAP (mg AAE/100 g)	Folin–Ciocalteau Reducing Capacity (mg GAE/100 g)	Cu^2+^ Chelating Activity (% Inhibition of the Cu^2+^-Pyrocatechol Violet Complex)	Fe^2+^ Chelating Activity (% inhibition of the Fe^2+^-Ferrozine Complex)
1	100	0	113 ± 0 ^b^	272 ± 13 ^b^	272 ± 7 ^b^	51 ± 1 ^b^	6 ± 1 ^c^
2	75	25	129 ± 2 ^a^	326 ± 2 ^a^	327 ± 3 ^a^	51 ± 2 ^b^	28 ± 7 ^b^
3	50	50	113 ± 2 ^b^	315 ± 10 ^a^	322 ± 22 ^a^	60 ± 1 ^a^	53 ± 3 ^a^
4	25	75	102 ± 6 ^c^	285 ± 3 ^b^	326 ± 2 ^a^	46 ± 1 ^c^	53 ± 8 ^a^
5	0	100	29 ± 1 ^d^	133 ± 2^c^	205 ±11 ^c^	20 ± 1 ^d^	31 ± 2 ^b^
*p-*Value (homoscedasticity)			0.145	0.322	0.443	0.189	0.445
*p-*Value (one-way ANOVA)			<0.001	<0.001	<0.001	<0.001	0.04

Different letters (^a–d^) in the same column represent statistically significant results (*p* ≤ 0.05). AAE = ascorbic acid equivalents; GAE = gallic acid equivalents.

**Table 5:** Antimicrobial activity of lyophilized marigold extract (*C. officinalis* L.) obtained with water and ethyl alcohol (50:50 v/v).

Micro-Organism	Inhibition Halo (cm)		
	Positive Control ^a^	Marigold Extract	*p*-Value ^b^
*L. monocytogenes* (ATCC 7644)	3.34 ± 1.04	1.04 ± 0.09	<0.01
*P. aeruginosa* (IAL 1853)	2.85 ± 1.63	1.07 ± 0.37	<0.01
*S.* Typhimurium (IAL 2431)	2.30 ± 0.61	0.17 ± 0.14	<0.01
*S.* Enteritidis (S 2887)	2.70 ± 1.56	0.19 ± 0.12	<0.01
*B. cereus* (IAL 55)	0.85 ± 0.31	0.34 ± 0.19	<0.01
*E. coli* (IAL 2064)	2.50 ± 1.17	1.45 ± 0.17	0.02
*S. aureus* (ATCC 13565)	0.80 ± 0.61	0.27 ± 0.14	0.02
*S. cerevisiae* (NCYC 1006)	3.19 ± 0.16	NI	-

NI = extract did not inhibit the growth. **^a^** Positive control was performed with ampicillin (10 µg/mL) for all bacterial strains, except *S. cerevisiae* was cicloheximide (1 μg/mL). Negative control was performed with sterile water for all microorganisms tested. ^b^ Statistical differences were obtained using unpaired Student *t* test (*p* ≤ 0.05).

**Table 6:** Total phenolic content and chemical antioxidant activity of organic yogurt with different concentrations of lyophilized marigold extract (*C. officinalis* L.).

Parameters	Formulations (g Lyophilized Marigold Extract/100 g Yogurt)					
	0	0.25	0.50	1.0	1.5	*p*-Value ^1^
Total phenolic content (mg GAE/100 g)	4.4 ± 0.1 ^e^	7.5 ± 0.1 ^d^	12.7 ± 0.1 ^c^	21.4 ± 0.3 ^b^	26.2 ± 0.4 ^a^	<0.001
DPPH (mg AAE/100 g)	0.4 ± 0.1 ^e^	3.3 ± 0.6 ^d^	5.8 ± 0.2 ^c^	9.3 ± 0.1 ^b^	12.9 ± 0.6 ^a^	<0.001
Total reducing capacity (mg QE/100 g)	ND	13.7 ± 0.4 ^d^	21.0 ± 0.7 ^c^	43.5 ± 0.3 ^b^	54.1 ± 1.0 ^a^	<0.001

ND = not detected. ^1^ Probability values obtained by one-way ANOVA. Different letters (^a–e^) in the same row represent statistical differences between yogurt formulations (*p* ≤ 0.05). AAE = ascorbic acid equivalents; GAE = gallic acid equivalents; QE = quercetin equivalents.

**Table 7:** Proximate composition, physicochemical characteristics, instrumental color, and texture of organic yogurts with and without the lyophilized marigold extract (LME).

Parameters	Organic Yogurt with LME ^1^	Organic Yogurt without Extract ^2^	*p*-Value ^3^
*Proximate composition*			
Carbohydrates (g/100 g)	7.84 ± 0.77	11.66 ± 0.56	0.002
Proteins (Nx6.38; g/100 g)	4.10 ± 0.04	4.13 ± 0.18	0.822
Lipids (g/100 g)	10.10 ± 0.39	10.57 ± 0.68	0.363
Ash (g/100 g)	0.78 ± 0.03	0.80 ± 0.17	0.856
Moisture (g/100 g)	76.99 ± 0.38	73.11 ± 0.95	0.002
Energy (kcal/100 g)	139.47 ± 3.73	158.32 ± 1.76	0.001
*Physicochemical characteristics*			
pH	5.05 ± 0.01	4.27 ± 0.01	<0.001
Titratable acidity (g lactic acid/100 g)	0.78 ± 0.01	0.79 ± 0.02	0.440
*Instrumental color*			
L* (lightness)	86.35 ± 1.29	93.54 ± 0.59	<0.001
Coordinate a* (redness)	1.34 ± 0.20	-0.35 ± 0.08	<0.001
Coordinate b* (yellowness)	17.16 ± 0.07	14.04 ± 0.18	<0.001
*Instrumental texture*			
Hardness (g)	22.20 ± 0.11	22.27 ± 0.17	0.562
Consistency (g/s)	322.40 ± 1.13	322.46 ± 2.32	0.969

^1^ Organic yogurt with 0.25 g of lyophilized marigold extract in 100 g of yogurt. ^2^ Natural organic yogurt (control—without lyophilized marigold extract). ^3^ Statistical differences were obtained using unpaired Student *t* test (*p* ≤ 0.05).

## Supplementary Materials

The following are available online at https://www.mdpi.com/2076-3921/8/11/559/s1, Figure S1: HPLC-DAD chromatogram of the *C. officinalis* flower extracted with water and ethyl alcohol (50:50 v/v): 1 = caffeic acid, 2 = *p*-coumaric acid, 3 = ferulic acid, 4 = procyanidin A2, 5 = quercetin-3-rutinoside, and 6 = ellagic acid, Figure S2: ESI-MS/MS chromatogram of the *C. officinalis* flower extracted with water and ethyl alcohol (50:50 v/v) and the tentative identification of phenolic compounds.
