# Supplementary material for: From the Field to the Pot: Phytochemical and Functional Analyses of Calendula officinalis L. Flower for Incorporation in an Organic Yogurt
Source: Antioxidants (Basel). 2019 Nov 15;8(11):559. doi: 10.3390/antiox8110559 (PMC6912323; doi:10.3390/antiox8110559)
Supplement: Supplementary file 1 [file antioxidants-08-00559-s001.pdf]

SUPPLEMENTARY MATERIAL

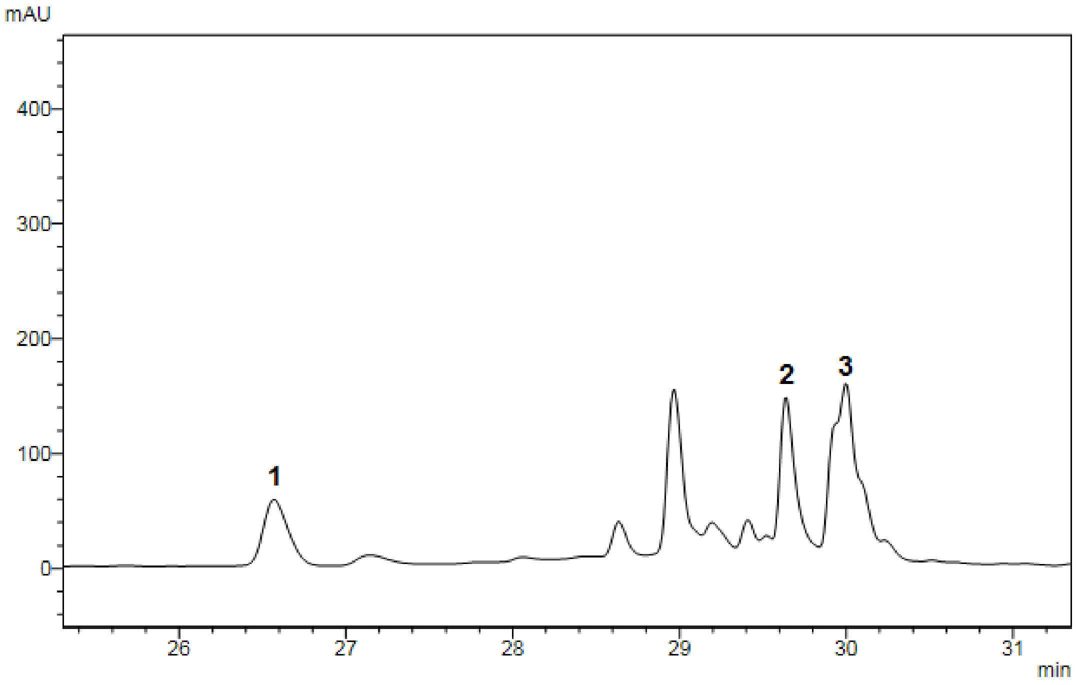

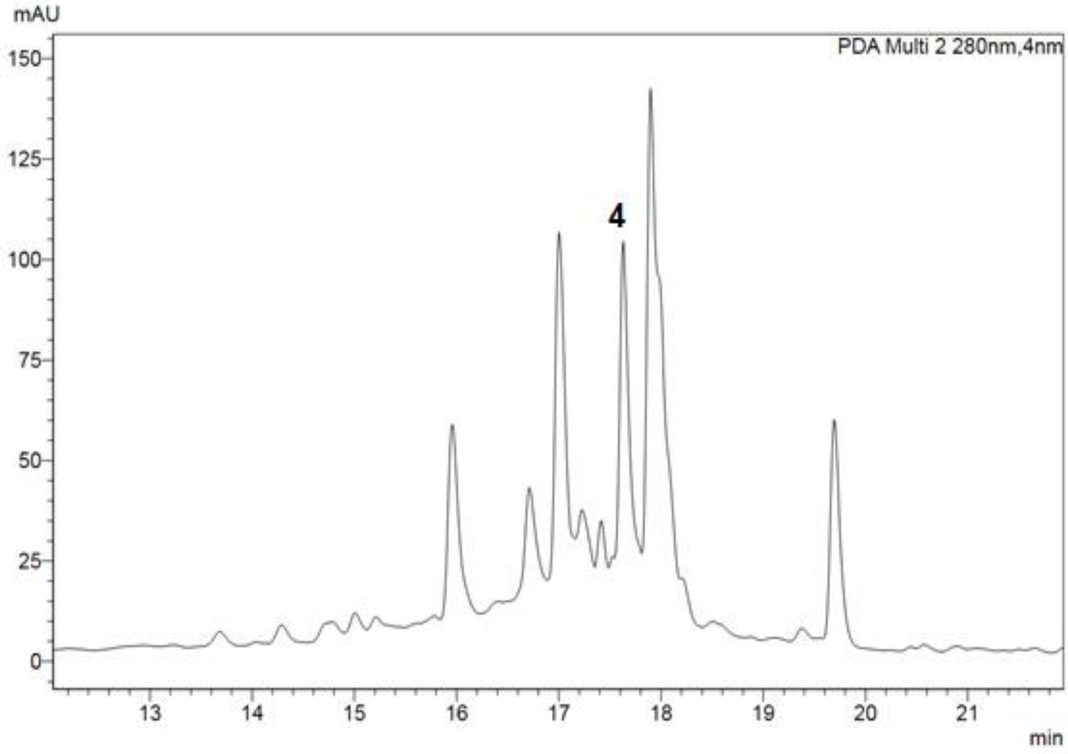

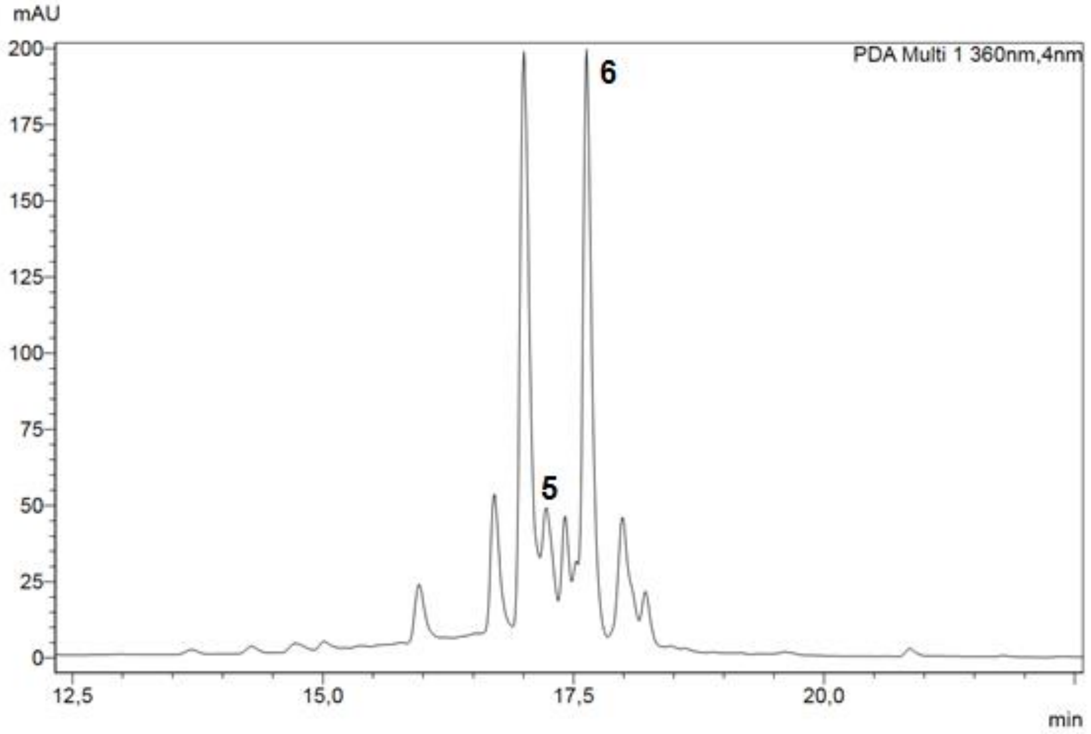

Figure S1. HPLC-DAD chromatogram of the *C. officinalis* flower extracted with water and ethyl alcohol (50:50 v/v): 1 = caffeic acid, 2 = *p*-coumaric acid, 3 = ferulic acid, 4 = procyanidin A2, 5 = quercetin-3-rutinoside, and 6 = ellagic acid.

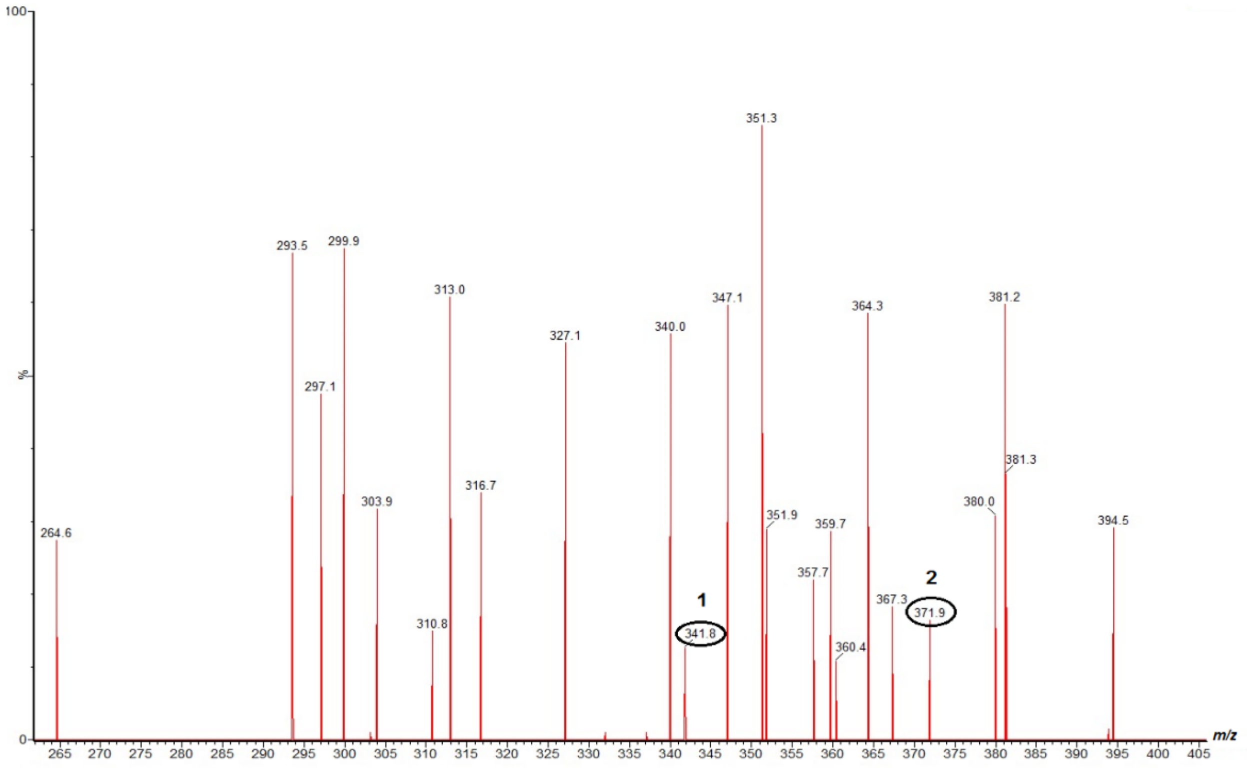
100
%
0
264.6
293.5
297.1
299.9
303.9
310.8
313.0
316.7
327.1
340.0
1
341.8
347.1
351.3
351.9
357.7
359.7
360.4
364.3
367.3
2
371.9
380.0
381.2
381.3
394.5
m/z
265 270 275 280 285 290 295 300 305 310 315 320 325 330 335 340 345 350 355 360 365 370 375 380 385 390 395 400 405

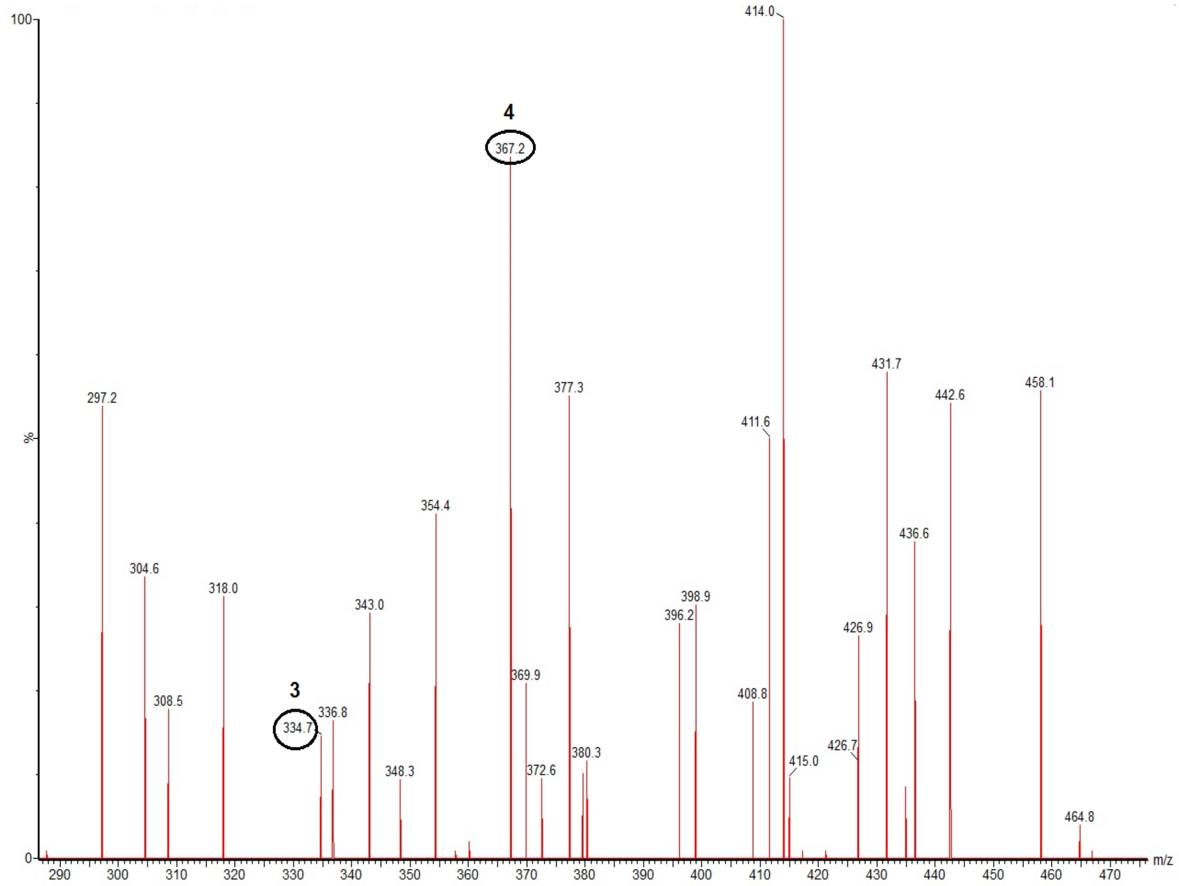
100
%
0
297.2
304.6
308.5
318.0
3
334.7
336.8
343.0
348.3
354.4
4
367.2
369.9
372.6
377.3
380.3
396.2
398.9
408.8
411.6
414.0
415.0
426.7
426.9
431.7
436.6
442.6
458.1
464.8
m/z
290 300 310 320 330 340 350 360 370 380 390 400 410 420 430 440 450 460 470

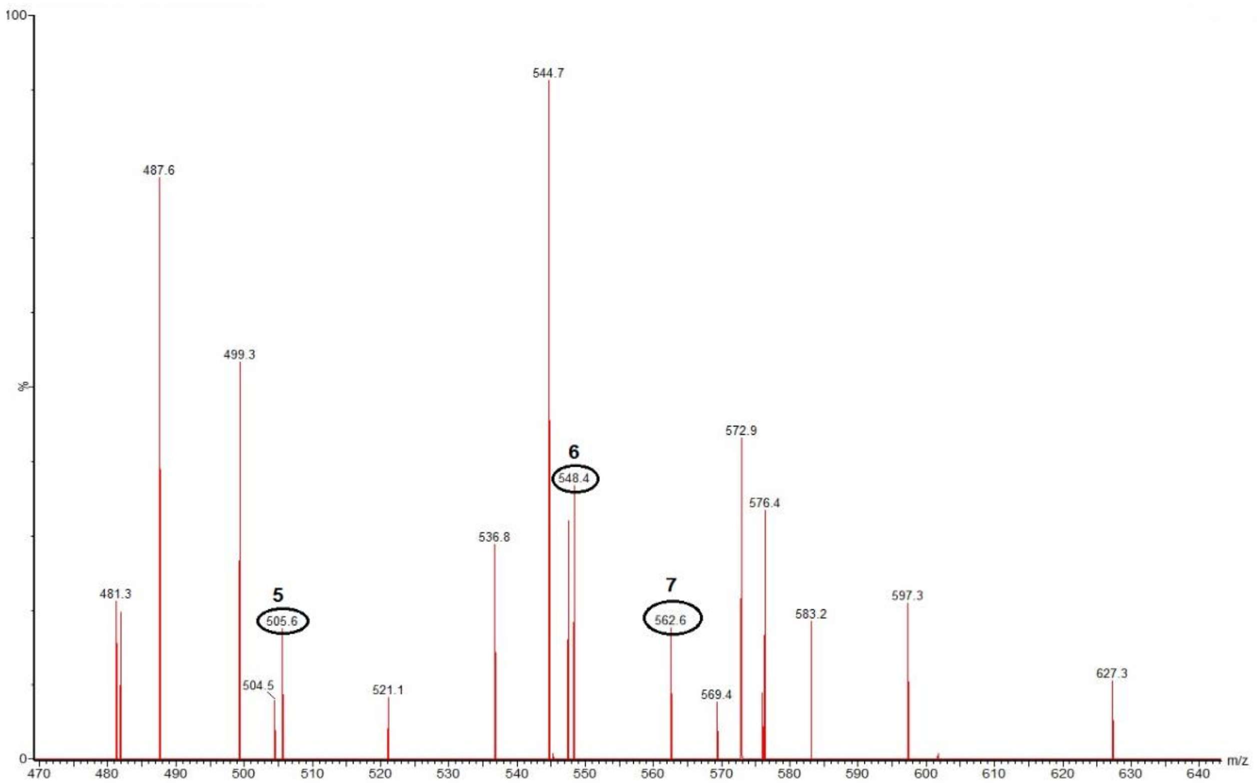

100
%
0
544.7
487.6
499.3
6
548.4
572.9
576.4
536.8
481.3
5
505.6
7
562.6
583.2
597.3
504.5
521.1
569.4
627.3
470
480
490
500
510
520
530
540
550
560
570
580
590
600
610
620
630
640
m/z

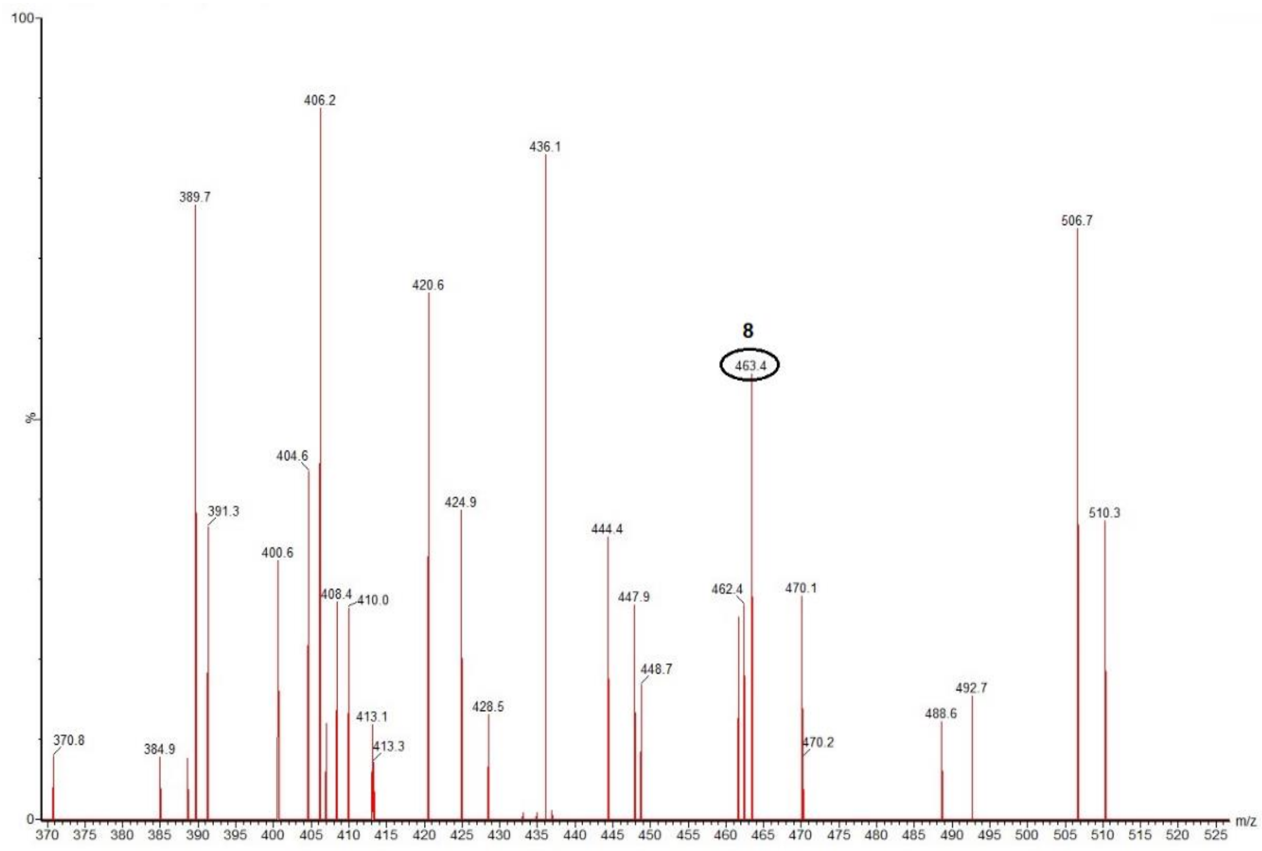

100
%
0
m/z
370.8
384.9
389.7
391.3
400.6
404.6
406.2
408.4
410.0
413.1
413.3
420.6
424.9
428.5
436.1
444.4
447.9
448.7
462.4
8
463.4
470.1
470.2
488.6
492.7
506.7
510.3

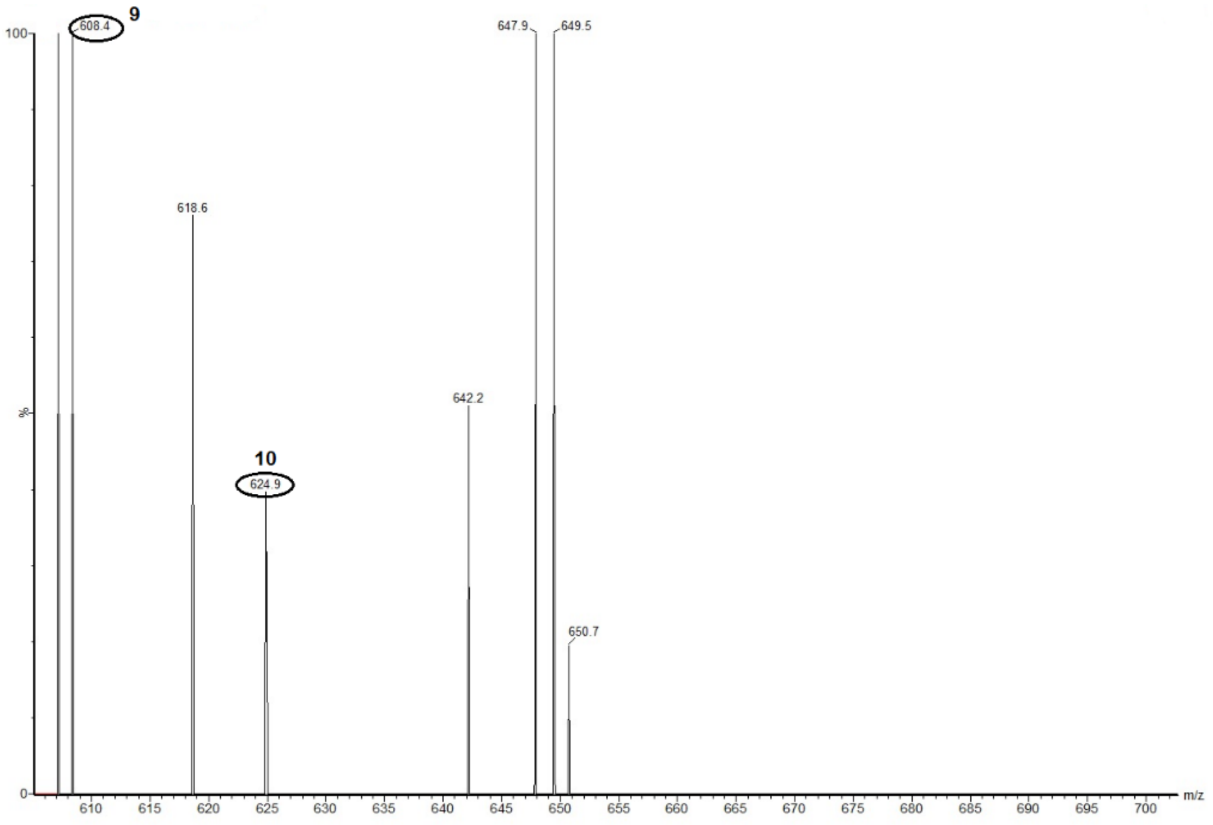

100
%
0
m/z
608.4
9
618.6
10
624.9
642.2
647.9
649.5
650.7

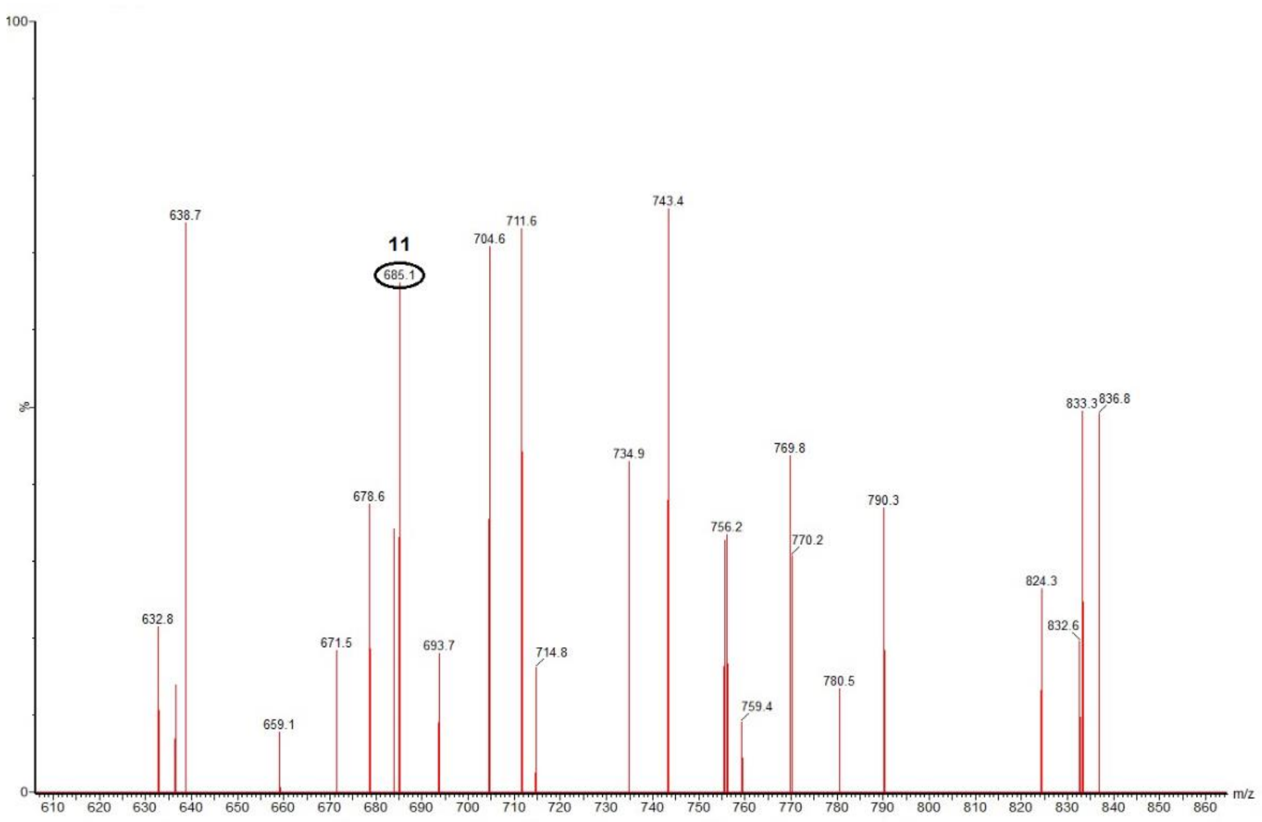

100
%
0
11
685.1
638.7
632.8
659.1
671.5
678.6
693.7
704.6
711.6
714.8
734.9
743.4
756.2
759.4
769.8
770.2
780.5
790.3
824.3
832.6
833.3
836.8
m/z

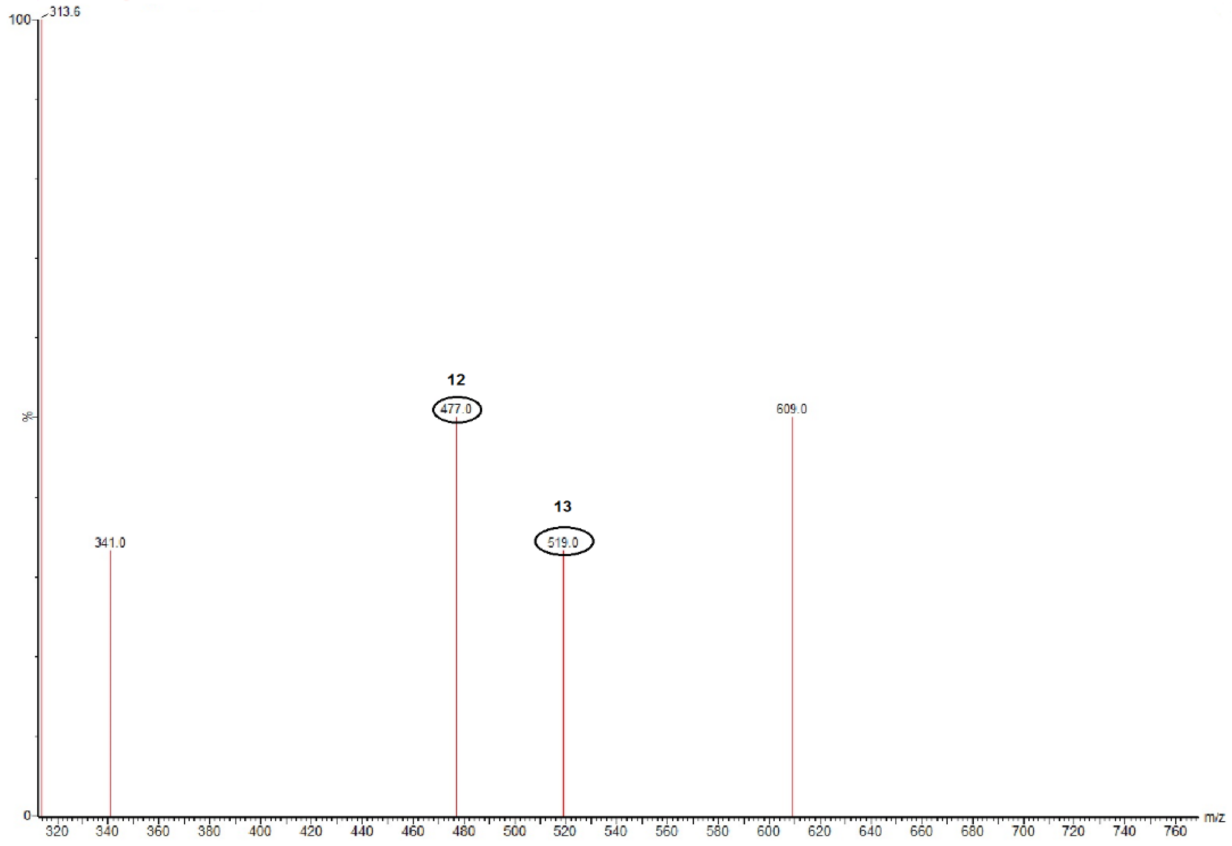

100
%
0
313.6
341.0
12
477.0
13
519.0
609.0
m/z

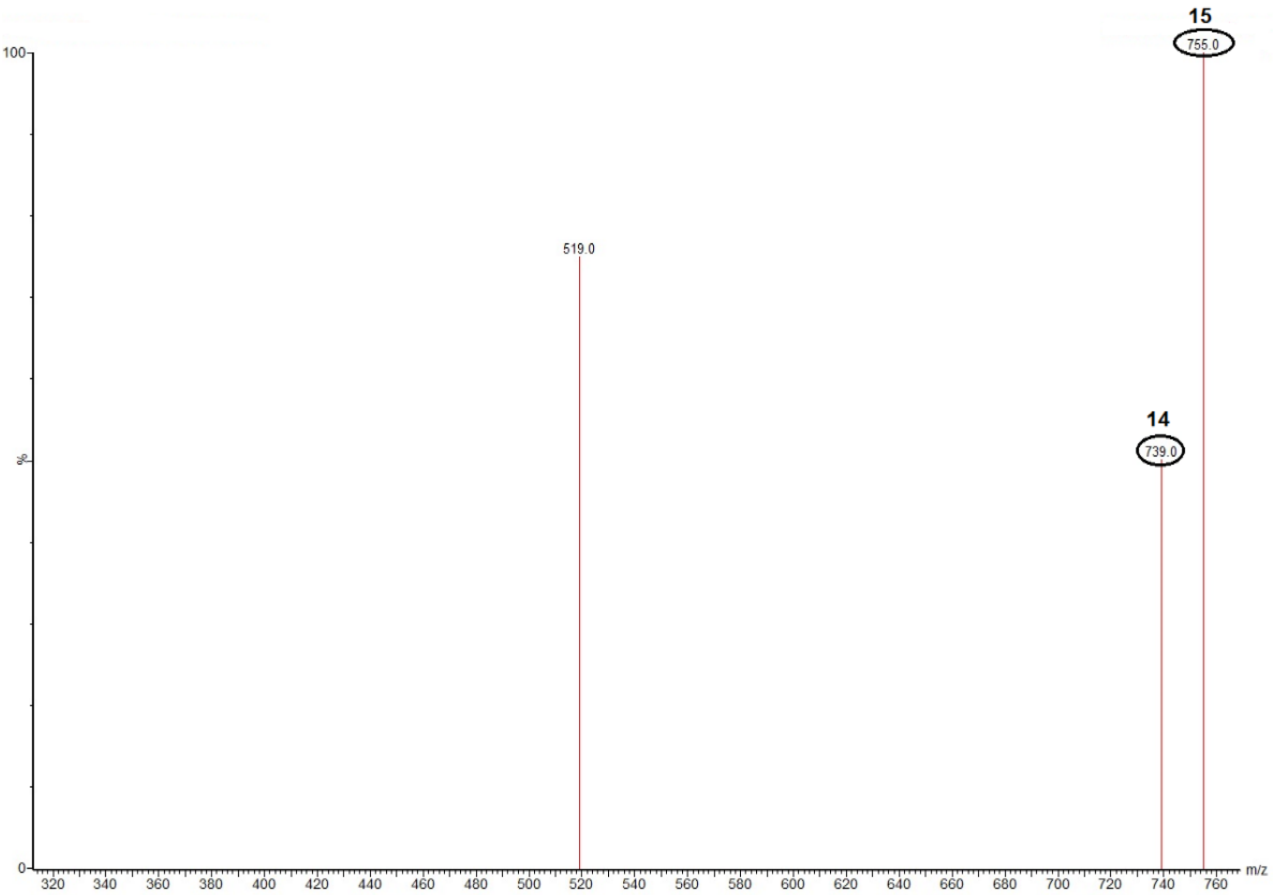
15
755.0
14
739.0
519.0
100
%
0
m/z
320 340 360 380 400 420 440 460 480 500 520 540 560 580 600 620 640 660 680 700 720 740 760

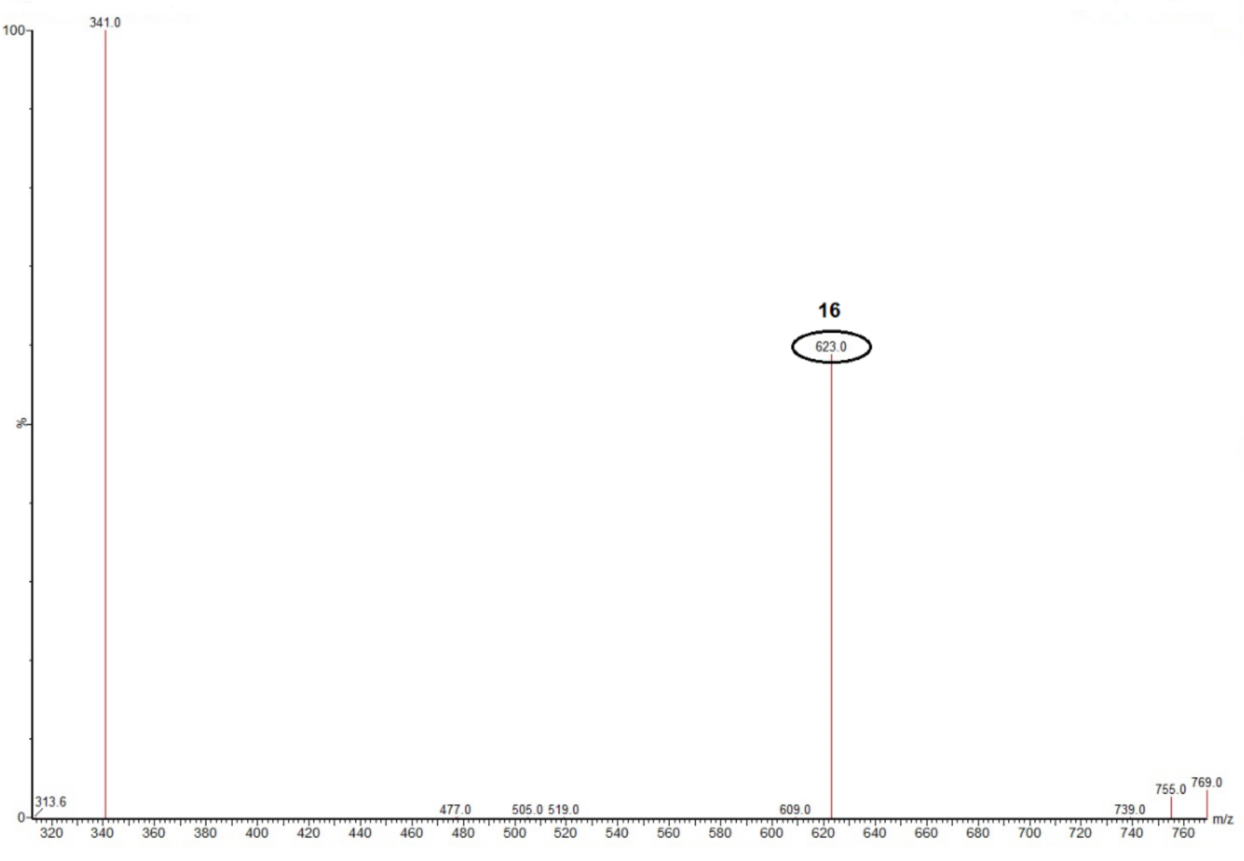
341.0
16
623.0
313.6
477.0
505.0 519.0
609.0
739.0
755.0
769.0
100
%
0
m/z
320 340 360 380 400 420 440 460 480 500 520 540 560 580 600 620 640 660 680 700 720 740 760

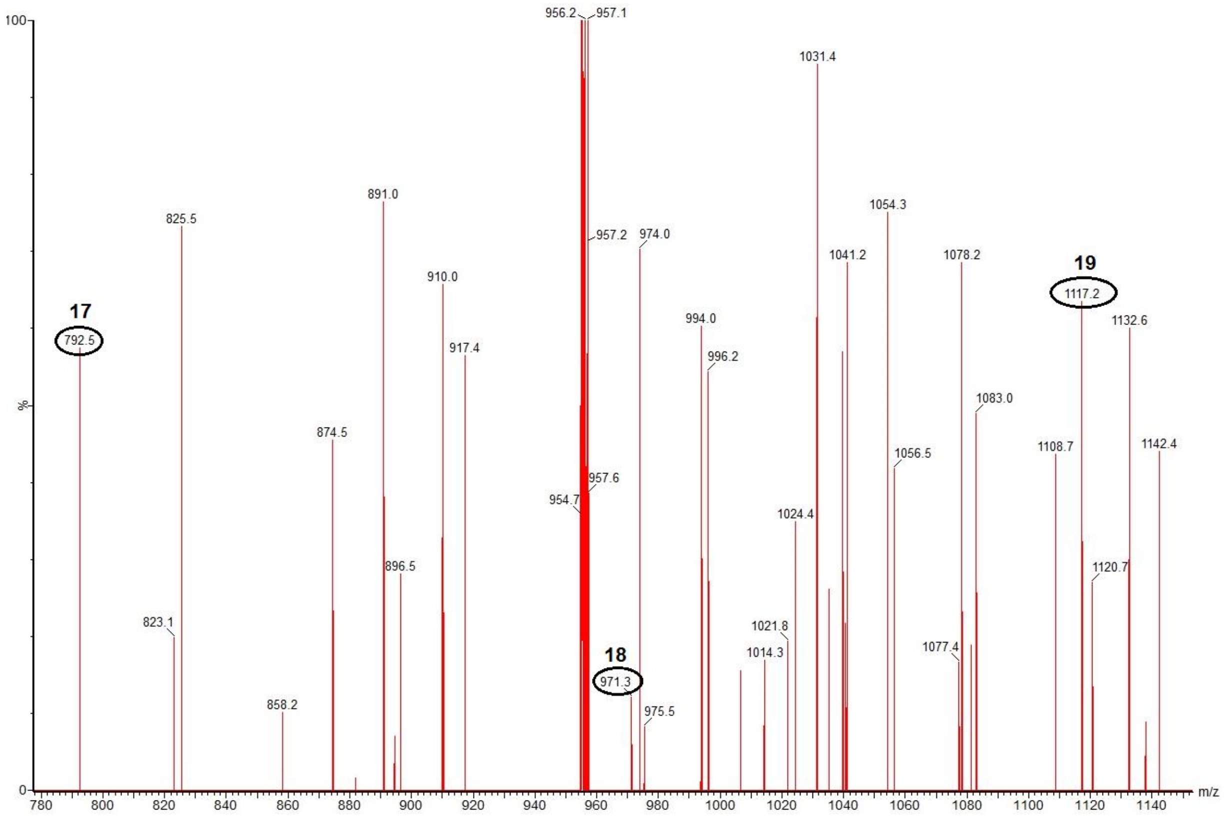

Figure S2. ESI-MS/MS chromatogram of the *C. officinalis* flower extracted with water and ethyl alcohol (50:50 v/v) and the tentative identification of phenolic compounds. Note: **1** = caffeic acid hexoside (*m/z* 341), **2** = isomeric form of hydroxyferulic acid hexoside (*m/z* 371), **3** = caffeoylshikimic acid (*m/z* 335), **4** = 5-*O*-feruloylquinic acid (*m/z* 367), **5** = quercetin derivative (*m/z* 505), **6** = quercetin-3-*O*-malonylhexoside (*m/z* 549), **7** = apigenin derivative (*m/z* 563), **8** = quercetin derivative (*m/z* 463), **9** = quercetin derivative (*m/z* 609), **10** = quercetin dihexoside (*m/z* 625), **11** = ligstroside hexoside (*m/z* 685), **12** = isorhamnetin derivative (*m/z* 477), **13** = isorhamnetin derivative (*m/z* 519), **14** = kaempferol-rhammosyrutinoside (*m/z* 739), **15** = quercetin-3-*O*-rhamnosylrutinoside (*m/z* 755), **16** = isorhamnetin derivative (*m/z* 623), **17** = calenduloside G (*m/z* 793), **18** = calendasaponin B (*m/z* 971), **19** = calendasaponin A (*m/z* 1117). Comparison with the reported MS/MS data obtained by Miguel et al. [44] and Faustino et al. [45].
